# Phylogenetic and Pathogenic Analysis of H5N1 and H5N6 High Pathogenicity Avian Influenza Virus Isolated from Poultry Farms (Layer and Broiler Chickens) in Japan in the 2023/2024 Season

**DOI:** 10.3390/v16121956

**Published:** 2024-12-20

**Authors:** Hayate Nishiura, Asuka Kumagai, Junki Mine, Yoshihiro Takadate, Saki Sakuma, Ryota Tsunekuni, Yuko Uchida, Kohtaro Miyazawa

**Affiliations:** Emerging Virus Group, Division of Zoonosis Research, National Institute of Animal Health, National Agriculture and Food Research Organization, Tsukuba 3050856, Japan; nishiurah840@affrc.go.jp (H.N.); kumagaia412@affrc.go.jp (A.K.); minejun84032@affrc.go.jp (J.M.); takadatey851@affrc.go.jp (Y.T.); sakumas438@affrc.go.jp (S.S.); tune@affrc.go.jp (R.T.); uchiyu@affrc.go.jp (Y.U.)

**Keywords:** high pathogenicity avian influenza virus, poultry farms, whole genome sequence, H5N1, H5N6, Japan, 2023/2024 season, phylogenetic analysis, pathogenic analysis

## Abstract

During the 2023–2024 winter, 11 high pathogenicity avian influenza (HPAI) outbreaks caused by clade 2.3.4.4b H5N1 and H5N6 HPAI viruses were confirmed in Japanese domestic poultry among 10 prefectures (*n* = 10 and 1, respectively). In this study, we aimed to genetically and pathologically characterize these viruses. Phylogenetic analysis revealed that H5N1 viruses were classified into the G2d-0 genotype, whereas the H5N6 virus was a novel genotype in Japan, designated as G2c-12. The G2c-12 virus shared PB2, PB1, PA, HA, and M genes with previous G2c viruses, but had NP and NS genes originating from avian influenza viruses in wild birds abroad. The N6 NA gene was derived from an H5N6 HPAI virus that was different from the viruses responsible for the outbreaks in Japan in 2016–2017 and 2017–2018. Experimental infections in chickens infected with H5N1(G2d-0) and H5N6(G2c-12) HPAI viruses showed no significant differences in the 50% chicken lethal dose, mean death time, or virus shedding from the trachea and cloaca, or in the histopathological findings. Different genotypes of the viruses worldwide, their introduction into the country, and their stable lethality in chickens may have triggered the four consecutive seasons of HPAI outbreaks in Japan.

## 1. Introduction

In 1996, an outbreak of the high pathogenicity avian influenza (HPAI) subtype H5, A/goose/Guangdong/1/1996 (Gs/Gd), was reported in China [1]. Since then, the Gs/Gd H5Nx HPAI lineage has been a threat to the poultry industry worldwide [2]. These progeny viruses have evolved rapidly and are composed of 10 divergent hemagglutinin (HA) clades (0–9) and several subclades [3]. The clade 2.3.4.4 of HPAI viruses emerged in China in 2014 and began to dominate global outbreaks. According to the World Health Organization, it is divided into eight subclades (a–h), of which subclade 2.3.4.4b is responsible for the largest number of HPAI epidemics worldwide [4]. Clade 2.3.4.4b HPAI viruses were first reported as the H5N8 subtype in early 2014. They were then further reassorted with Eurasian low pathogenicity avian influenza viruses and disseminated from Siberia for detection in a wide range of areas, including Europe, Africa, the Middle East, and Asia [5]. This resulted in the emergence of the H5N1 virus in clade 2.3.4.4b in the 2020s. H5N1 reassortment has caused huge outbreaks in various wild bird species, including seabirds, on all continents except Australia, along with economic losses in the poultry industry [6].

The first outbreak caused by clade 2.3.4.4b HPAI in Japan was reported in the 2017/18 winter season [7]. Following the outbreak caused by the H5N6 subtype virus, the 2020/21 season outbreaks were caused by the H5N8 subtype virus. Since then, HPAI outbreaks caused by the H5Nx subtype viruses of clade 2.3.4.4b have been confirmed in Japan for three consecutive seasons [8,9,10,11]. Based on a detailed phylogenetic analysis of the HA gene, H5Nx viruses isolated in Japan were divided into two genetic groups: G1 and G2 [12,13]. The main genetic group that caused the recent outbreaks in Japan was G2 (those circulating in Europe in late 2020). The G2 group was further divided into sub-genetic groups G2a, G2b, G2c, and G2d [11,14,15,16]. The neuraminidase (NA) subtypes of the HPAI viruses also varied.

During the winter of 2023–2024 in Japan, 11 outbreaks of HPAI (10 H5N1 and 1 H5N6) were identified on poultry premises in 10 prefectures. The first HPAI outbreak was reported on 24 November 2023, and the last was reported on 28 April 2024. During this period, approximately 856,000 poultry were culled to prevent and control HPAI outbreaks.

In this study, we genetically characterized the H5N1 and H5N6 subtype viruses that caused HPAI outbreaks in Japan during the 2023/2024 winter season using phylogenetic analyses for all eight segments, and investigated the virological characteristics of the representative isolates of both subtypes by the experimental infection of chickens.

## 2. Materials and Methods

### 2.1. Virus Isolation and Whole-Genome Sequencing

For virus isolation, tracheal or cloacal swabs from poultry premises that tested positive using a rapid diagnostic kit (Fujirebio Inc., Tokyo, Japan) and were suspected of HPAI virus infection were inoculated into embryonated chicken eggs at the diagnostic laboratories of the Animal Health Service Centers in each prefecture. At the National Institute of Animal Health (NIAH), Japan, we obtained allantoic fluid inoculated with swab samples that showed HA activity, based on Japanese pathological appraisal guidelines. Whole-genome sequences of the viruses were obtained using MiSeq (Illumina, San Diego, CA, USA) next-generation sequencing technology, the details of which have been reported previously [13]. Two isolates were collected during each outbreak. Briefly, cDNA libraries were prepared using the NEBNext Ultra II RNA Library Prep Kit for Illumina (New England Biolabs, Ipswich, MA, USA), and sequences were determined using the MiSeq Reagent Kit v. 2 (Illumina). The obtained sequence data were reconstructed into a complete consensus sequence using CLC Genomics Workbench (v. 23.0.2, Qiagen, Hilden, Germany) and FluGAS software (v. 2.5, World Fusion, Tokyo, Japan). Finally, we registered the newly identified viral sequences in the Global Initiative on Sharing All Influenza Data (GISAID) (http://platform.gisaid.org (accessed on 15 January 2024)) with accession numbers (Table S1). To construct the multiple sequence alignment, the nucleotide sequences of the avian influenza viruses (AIVs) were downloaded from GISAID (accessed on 18 June 2024). The loaded isolates sequenced with our 22 new isolates were aligned in MAFFT version 7.526 [17] and edited using BioEdit v. 7.2.5 [18]. For phylogenetic analyses, sequences with ambiguous nucleotide bases were removed, leaving 36,365 sequences for polymerase basic protein (PB) 2 genes; 35,420 for PB1; 38,084 for polymerase acidic protein (PA); 18,779 for H5; 38,436 for nucleoprotein (NP); 13,858 for N1; 4504 for N6; 38,394 for matrix protein (MP); and 32,145 for nonstructural protein (NS). Maximum likelihood trees were constructed using FastTree v. 2.1.11 software [19] based on each gene of the multiple sequence alignment.

### 2.2. Animal Experiments

The following three HPAI viruses were used in the animal experiments: A/chicken/Saga/23A2T/2023 (Saga/23A2T, H5N1) and A/chicken/Kagoshima/23B1T/2024 (Kagoshima/23B1T, H5N6) as representative isolates of the current season, and A/chicken/Hokkaido/22B3C/2022 (Hokkaido/22B3C, H5N1) as a comparison isolate from the previous season. The experiments were approved by the Institutional Committee for Ethics of Animal Experiments (approved number R4-I014-NIAH-5) and conducted in biosafety level 3 (BSL3) facilities at NIAH. Animal experiments were designed as previously reported [8,9,10,11]. Fifty-five specific-pathogen-free White Leghorn chickens (four-week-old) were purchased from Nisseiken Co., Ltd. (Tokyo, Japan). Serum was collected from all birds before virus inoculation and ELISA was performed using the IDEXX Influenza A Ab Test kit (IDEXX Laboratories, Westbrook, ME, USA) to confirm that all chickens were serologically negative for influenza A virus. To evaluate the 50% lethal dose (CLD_50_), chickens housed in the isolator were randomly divided into eleven groups of five chickens each, with *ad libitum* access to food and water. Each group was intranasally inoculated with 100 µL of Saga/23A2T at doses ranging from 10^4^ to 10^6^ 50% egg infectious dose (EID_50_) or Kagoshima/23B1T and Hokkaido/22B3C at doses ranging from 10^3^ to 10^6^ EID_50_ per chicken. The mean death time (MDT) of infected chickens was calculated from the results of the 10^6^ EID_50_-inoculated group. We clinically monitored all chickens and collected tracheal and cloacal swabs from chickens in the 10^6^ EID_50_ inoculated groups at 1, 2, 3, 5, 7, 10, and 14 days post-inoculation (dpi), or when the chickens died. The infected chickens were euthanized by the intravascular injection of an overdose of sodium pentobarbital at the end of the experiment, or when the humane endpoint was reached. The collected swabs were suspended in 2 mL minimum essential medium (Thermo Fisher Scientific, Waltham, MA, USA) containing 0.5% bovine serum albumin (Sigma-Aldrich, St. Louis, MO, USA), 25 mg/mL amphotericin B (Gibco, Life Technologies, Grand Island, NY, USA), 1000 units/mL penicillin (Gibco), 1000 mg/mL streptomycin (Gibco), 0.01 M HEPES (Gibco), and sodium bicarbonate solution (Gibco), and stored at −80 °C until use. The swab fluids were inoculated into 9–11-day-old embryonated eggs to measure the infectious viral titer, and the EID_50_ was calculated using the Reed–Muench method [20]. The detection limit was 0.2 log EID_50_/mL. At the end of the experiment, serum was collected from surviving chickens and tested for infection. The presence of antibodies against the influenza A virus in the serum was examined using the method described above.

### 2.3. Histopathology

Pathological examinations were performed under BSL3 conditions. Tissue samples were collected from carcasses with minimal autolysis, shortly after death or humane euthanasia. The samples collected included the liver, spleen, kidney, heart, lungs, intestines, pancreas, thymus, bursa of Fabricius, and brain. They were fixed in 10% neutral-buffered formalin, processed routinely, embedded in paraffin wax, sectioned at a 3–5 µm thickness, and stained with hematoxylin and eosin. Immunostaining was performed using an antibody-labeled polymer method to detect viral antigens. Endogenous peroxidase activity in sections was blocked using 3% hydrogen peroxide in ultrapure water. The antigen retrieval step involved digestion using 1 mg/mL of actinase E (Kaken Pharmaceutical Co., Tokyo, Japan) in phosphate-buffered saline (PBS) for 10 min at 37 °C. The sections were blocked with PBS containing 5% skim milk (Fujifilm Wako Pure Chemicals Co., Osaka, Japan). The sections were then incubated overnight at 4 °C with mouse monoclonal antibodies specific for the nucleoprotein of influenza virus A (1:1000; HYB 340-05; Statens Serum Institut, Copenhagen, Denmark). The Histofine Simple Stain MAX-PO(M) kit (Nichirei Bioscience, Tokyo, Japan) was used for the secondary antibody. Immunoreactivity was determined using 3,3′-diaminobenzidine. The immunohistochemical scoring of major organs was performed based on a previous study [21].

## 3. Results

### 3.1. HPAI Outbreaks in Japan During the 2023/2024 Winter Season

**Figure 1:**
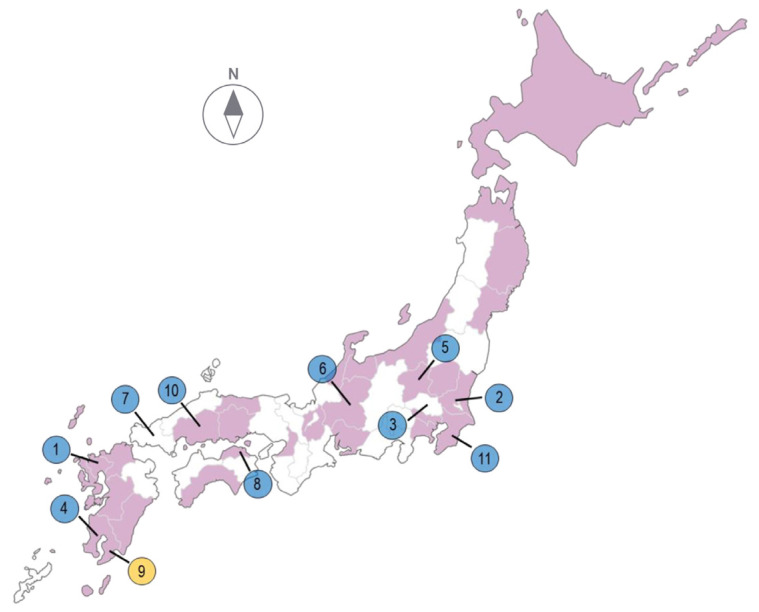
Geographic locations of prefectures where H5-subtype high pathogenicity avian influenza (HPAI) cases were confirmed in Japan during the winter of 2023–2024. The blue circles indicate the HPAI cases caused by H5N1 HPAI virus and the yellow circle indicates the HPAI case caused by the H5N6 HPAI virus. The order of outbreaks is indicated by the number in each circle. The purple areas represent prefectures where the H5-subtype HPAI virus has been detected in wild birds.

From 11 November 2023 to 28 April 2024, there were 10 outbreaks due to H5N1 subtype HPAI virus and there was 1 outbreak due to H5N6 subtype HPAI virus in 10 prefectures in Japan. All outbreaks were reported in areas of Japan that were not located in the northern part of the country (Figure 1 and Table S1). During the same period, 143 HPAI virus-positive cases were identified in samples from dead wild birds throughout Japan, including northern Honshu and Hokkaido (Figure 1). The detailed information on the outbreaks of the current season is based on a Self-Declaration submitted to the World Organization for Animal Health (WOAH) by the Ministry of Agriculture, Forestry and Fisheries of Japan (https://www.woah.org/app/uploads/2024/06/2024-06-japan-hpai-eng2.pdf (accessed on 6 December 2024)).

### 3.2. Genetic Characteristics of H5 Subtype HPAI Viruses Isolated from Poultry in Japan During the Winter of 2023–2024

#### 3.2.1. Phylogenetic Analysis of the HA Gene of H5 Subtype HPAI Viruses Isolated from Japanese Poultry Premises During the Winter of 2023–2024

**Figure 2:**
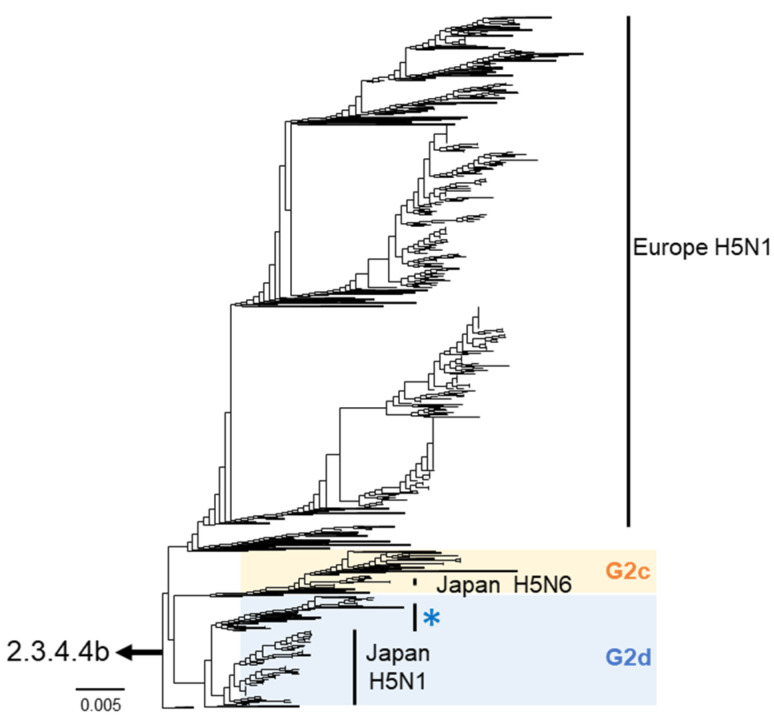
Phylogenetic trees based on the HA gene. Maximum-likelihood tree of clade 2.3.4.4b covering the G2c and G2d subgroups. The blue asterisk indicates the cluster including H5-subtype HPAI viruses isolated during the winter of 2022–2023. Europe H5N1 viruses isolated in the same season were genetically close to HPAI viruses isolated in Japan.

In all H5-subtype viruses isolated during the winter of 2023–2024, according to the manual of the WOAH (https://www.woah.org/fileadmin/Home/eng/Health_standards/tahm/3.03.04_AI.pdf (accessed on 6 December 2024)), genetic sequencing confirmed the presence of multiple basic amino acid residues in the HA cleavage site (PLREKRRKR/GLF), indicating the high pathogenicity of these isolates. Based on the HA gene phylogenetic analysis, all isolates belonged to the G2 group of clade 2.3.4.4b. Specifically, 10 H5N1 isolates were classified into the G2d subgroup, and one H5N6 isolate was classified into the G2c subgroup (Figure 2). Ten H5N1 isolates in the G2d subgroup showed an average nucleotide identity of 99.7% with the HA gene. Phylogenetic analysis of the HA gene also revealed that H5N1 viruses isolated in Europe during the same season shared a common ancestor with HPAI viruses isolated in Japan (both G2d and G2c; Figure 2).

#### 3.2.2. Genetic Characteristics of H5N1 HPAI Viruses of the G2d Subgroup Isolated from Poultry Premises During the Winter of 2023–2024

**Figure 3:**
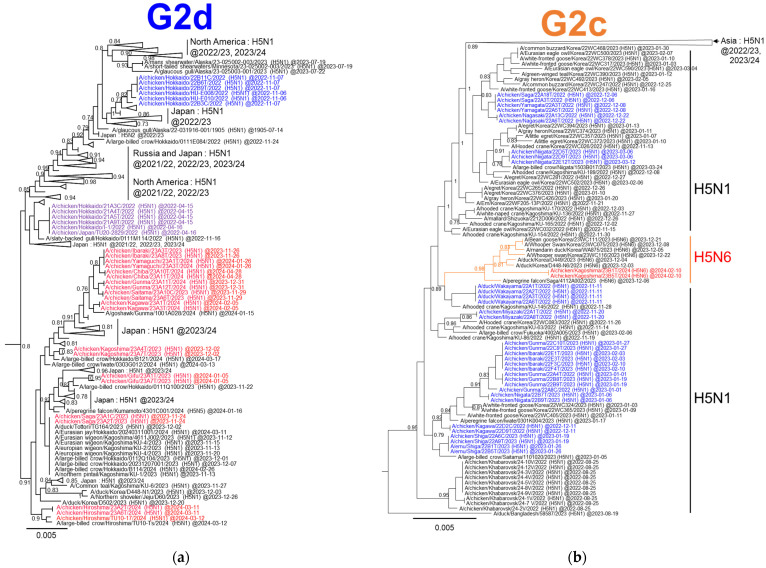
Detailed phylogenetic trees focusing on the HA gene. (**a**,**b**) Extended phylogenetic trees covering the H5-subtype HPAI viruses of the (**a**) G2d and (**b**) G2c subgroups. The purple-, blue-, and red-colored names refer to H5-subtype HPAI viruses isolated from poultry in Japan during the winters of 2021–2022, 2022–2023, and 2023–2024, respectively. More than 0.60 of Fast-global bootstrap values are shown.

A detailed phylogenetic tree based on the HA gene of the G2d subgroup shows that the H5N1 HPAI viruses isolated in Japan during the 2023/2024 winter season clustered with those isolated in Korea during the same winter season, and were genetically closest to those isolated from poultry in Japan during the 2021/2022 winter season from February to May 2022 (Figure 3a). In contrast, these viruses formed a slightly distant cluster from the H5N1 HPAI viruses isolated in Japan during the winter of 2022–2023. The phylogenetic analysis of the NA gene also showed that the H5N1 HPAI viruses isolated in both Japan and Korea during the winter of 2023–2024 possessed an N1 gene closely related to that of the H5N1 HPAI viruses isolated in Japan during the late winter of 2021–2022 (Figure S1e). Phylogenetic analyses based on six other internal genes (PB2, PB1, PA, NP, M, and NS) showed that all H5N1 HPAI viruses isolated in Japan during the winter of 2023–2024 had internal genes with the same relationship as HA and NA genes (Figure S1a–g). These results indicate that the genotype of H5N1 HPAI viruses isolated in Japan during the winter season is G2d-0, which is the main genotype of the G2d subgroup found in Japan for several seasons.

#### 3.2.3. Genetic Characteristics of H5N6 Virus of the G2c Subgroup Isolated from Poultry Premises During the Winter of 2023–2024

**Figure 4:**
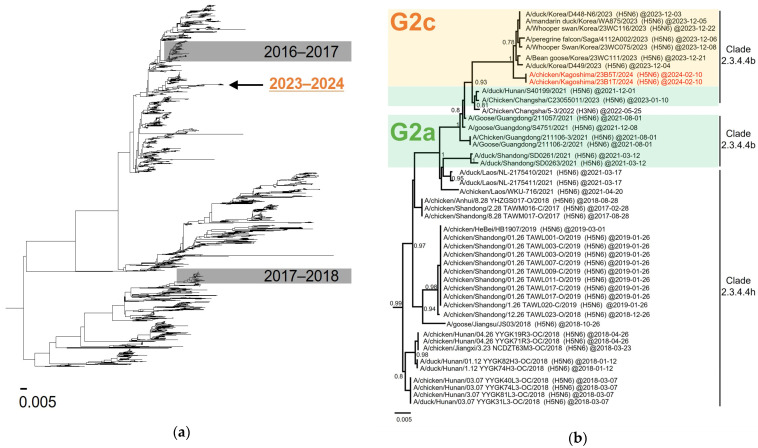
Phylogenetic trees focusing on the neuraminidase (NA) gene. (**a**) Maximum likelihood tree based on the N6 NA gene covering the H5N6 viruses causing HPAI outbreaks in Japan to date. (**b**) An extended phylogenetic tree of the N6 NA gene covering the Japanese H5N6 HPAI viruses of the G2c subgroups. The red-colored names represent H5N6 HPAI viruses isolated from poultry in Japan during the winter of 2023–2024. More than 0.60 of Fast-global bootstrap values are shown.

Figure 3b shows an enlarged phylogenetic tree focusing on HA in the G2c subgroup. The H5N6 HPAI isolated in Japan formed a cluster with the H5N6 HPAI isolated in Korea during the same winter season (Figure 3b). The HA genes of these H5N6 viruses shared a common ancestor with the H5N1 HPAI viruses reported in Japan during the winter of 2022–2023. The phylogenetic tree based on the N6 NA gene showed that the origin of the NA gene was different from that of the H5N6 HPAI viruses that caused HPAI outbreaks in Japan during the winter seasons of 2016–2017 and 2017–2018 (Figure 4a). The N6 NA gene of the Japanese and Korean H5N6 HPAI isolates during the winter of 2023–2024 was closely related to the H5N6 HPAI viruses of the G2a subgroup reported in Hunan and Guangdong provinces, China, in 2021 (Figure 4b). The N6 NA genes of the H5N6 HPAI viruses of the G2a and G2c subgroups branched from those of the H5N6 HPAI viruses belonging to clade 2.3.4.4h, which were isolated in Shandong, Jiangsu, and Hunan provinces in China, as well as from the H5N6 HPAI isolates in Laos (Figure 4b).

**Figure 5:**
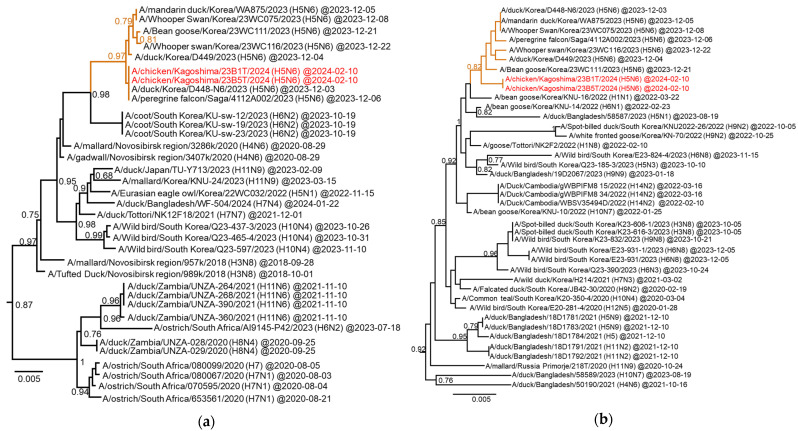
Phylogenetic trees focusing on the nucleoprotein (NP) and nonstructural protein (NS) genes. (**a,b**) Enlarged maximum likelihood trees based on the (**a**) NP gene and (**b**) NS gene covering the H5N6 HPAI viruses isolated in Japan and Korea during the winter of 2023–2024. The red-colored names represent H5N6 HPAI viruses isolated from poultry in Japan during the winter of 2023–2024. More than 0.60 of Fast-global bootstrap values are shown.

To determine the genotype of the Japanese H5N6 HPAI isolate, phylogenetic analyses were conducted based on internal genes. All the internal genes of the Japanese H5N6 isolate clustered with those of the Korean H5N6 HPAI isolates during the same winter season (Figure 5a,b and Figure S2). The PB2, PB1, PA, and MP genes of the Japanese H5N6 HPAI isolate were closely related to those of the H5N1 isolates of the G2c subgroup reported in the winter of 2022–2023 (Figure S2a–d). In contrast, the NP and NS genes of the Japanese H5N6 HPAI isolate were closely related to those of AIVs except for H5Nx HPAI viruses. The NP gene of the H5N6 HPAI isolate shared a common ancestor with that of the H6N2 virus isolated in Korea during early winter, and was closely related to the H4N6 AIV isolated in Novosibirsk in August 2020 (Figure 5a). Notably, the Korean H5N1 HPAI virus (A/Eurasian eagle owl/Korea/22WC032/2022) of the G2c subgroup, isolated in October 2022, has an NP gene genetically similar to the Japanese H5N6 HPAI virus. As shown in Figure 5b, the NS gene of the Japanese H5N6 HPAI isolate shared a common ancestor with that of the H1N1 AIV isolated in Korea in March 2022, and was closely related to those of AIVs possessing different HA genes reported in Korea, Japan, Bangladesh, and Cambodia, including non-GS/GD H5-subtype viruses, between 2022 and 2023. A genetically similar NS gene was detected in the H5N1 virus (A/duck/Bangladesh/58587/2023) from the G2c subgroup isolated in Bangladesh in August 2023. These results indicate that the Japanese H5N6 isolate is an H5-subtype HPAI virus with a novel set of gene segments (designated as genotype G2c-12).

### 3.3. Virological Characteristics of Saga/23A2T and Kagoshima/23B1T

**Figure 6:**
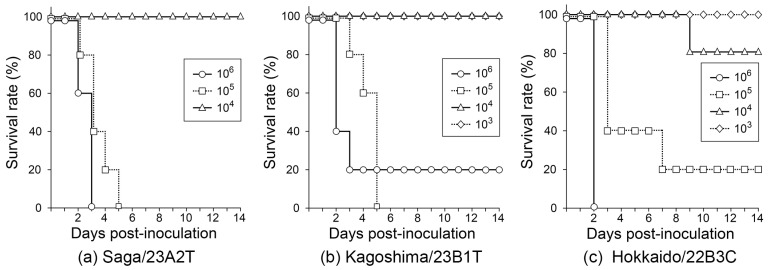
Survival rates of chickens inoculated with (**a**) Saga/23A2T, (**b**) Kagoshima/23B1T, and (**c**) Hokkaido/22B3C. Circles, squares, triangles, and rhombuses indicate the survival rates of chickens inoculated with 10^6^, 10^5^, 10^4^, and 10^3^ 50% egg infectious dose (EID_50_) of each virus, respectively.

In the present study, the following three different H5-subtype HPAI isolates were intranasally inoculated into chickens: Hokkaido/22B3C (H5N1, G2d-0 isolated in the 2022/2023 season), Saga/23A2T (H5N1, G2d-0 isolated in the 2023/2024 season), and Kagoshima/23B1T (H5N6, G2c-12 in the 2023/2024 season). All chickens inoculated with 10^6^ and 10^5^ EID_50_ Saga/23A2T died within 3 and 5 dpi, respectively. However, all chickens inoculated with 10^4^ EID_50_ survived (Figure 6a). Four out of five chickens inoculated with 10^6^ EID_50_ Kagoshima/23B1T died within 3 dpi, while one survived to 14 dpi, although all chickens in the 10^5^ EID_50_-inoculated group died by 5 dpi. No chickens died in the 10^4^ EID_50_ and 10^3^ EID_50_ inoculated groups (Figure 6b). In the Hokkaido/22B3C group, all chickens inoculated with 10^6^ EID_50_ died within 2 dpi, and one and four chickens in the 10^4^ and 10^5^ EID_50_-inoculated groups, respectively, survived the observation period (Figure 6c). Therefore, the 50% chicken lethal doses (CLD_50_) of Saga/23A2T, Kagoshima/23B1T, and Hokkaido/22B3C were calculated as 10^4.50^, 10^4.60^, and 10^4.50^ EID_50_, respectively (Table 1). The MDTs of the chickens inoculated with Hokkaido/22B3C, Saga/23A2T, and Kagoshima/23B1T at 10^6^ EID_50_ were calculated for 2.2 days (53 h), 2.7 days (64 h), and 2.3 days (54 h), respectively (Table 1). No significant clinical signs were observed in any of the chickens, except for some depressive signs in a few chickens inoculated with higher doses. No detectable antibodies against the influenza A virus were found in any of the sera collected from the surviving chickens on day 14 post-inoculation.

**Figure 7:**
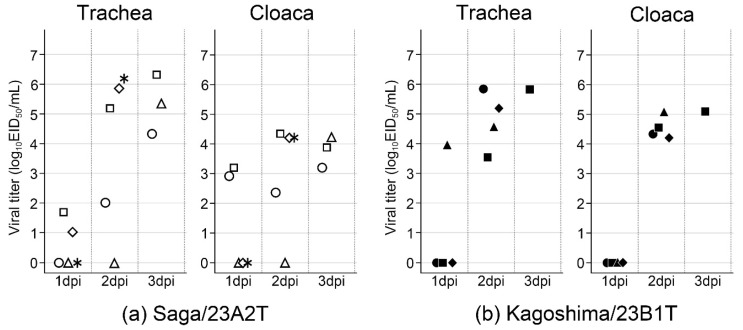
Kinetics of virus titers in each tracheal and cloacal swab from chickens inoculated with 10^6^ EID_50_ of (**a**) Saga/23A2T and (**b**) Kagoshima/23B1T. Circles, squares, triangles, rhombuses, and asterisks represent the different chickens.

To analyze viral shedding, tracheal and cloacal swabs were collected from chickens infected with 10^6^ EID_50_ of each virus. The kinetics of viral titers in the tracheal and cloacal swabs at 1, 2, and 3 dpi are shown in Figure 7. The mean peak virus titers for Saga/23A2T-infected chickens were 5.6 ± 0.4 log_10_EID_50_/mL in the tracheal swabs and 4.0 ± 0.2 log_10_EID_50_/mL in cloacal swabs (Table 1). For Kagoshima/23B1T-infected chickens, the mean peak virus titers in the tracheal and cloacal swabs were 5.4 ± 0.4 log_10_EID_50_/mL and 4.7 ± 0.2 log_10_EID_50_/mL, respectively (Table 1).

### 3.4. Histopathology of Chicken Infected with Saga/23A2T or Kagoshima/23B1T

Autopsies were performed shortly after death, with minimal autolysis of the carcass or humane euthanasia for accurate assessment. Some chickens had mild facial edema, but no other noticeable changes were observed. The gross lesions included the retention of pericardial fluid (hydropericardium), diffuse pulmonary congestion and edema, and friable, pale discoloration of the liver (Figure S3).

**Figure 8:**
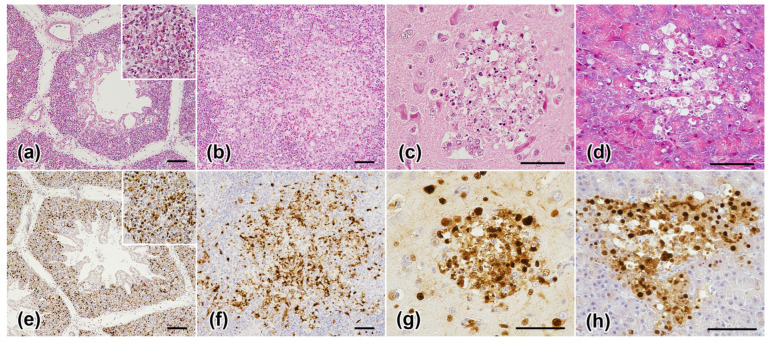
Histological and immunohistochemical findings in chickens inoculated with 10^6^ EID_50_ Saga/23A2T. (**a**) Lung: respiratory lobules. The air capillaries lose their pre-existing structure and are replaced by inflammatory cells, cellular debris, and fibrin (insert). Hematoxylin and eosin (HE) stain. Bar = 100 µm. (**b**) Spleen. Necrosis of ellipsoid with fibrinous exudation. HE stain. Bar = 50 µm. (**c**) Cerebrum. Focal necrosis of neurons and glial cells with pyknosis and karyorrhexis of the nucleus and neuropil vacuolation. HE stain. Bar = 50 µm. (**d**) Pancreas. Focal necrosis of acinar cells. HE stain. Bar = 50 µm. (**e**) Lung: respiratory lobules. Strong intranuclear immunohistochemical signals were found in degenerating, sloughed cells in air capillaries (inset). Immunolabeling for influenza A virus. Bar = 100 µm. (**f**) Spleen. There is positive nuclear and cytoplasmic staining of ellipsoidal cells. Immunolabeling for influenza A virus. Bar = 50 µm. (**g**) Cerebrum. Nuclei of neurons and glial cells are positive for influenza A virus. Immunolabeling for influenza A virus. Bar = 50 µm. (**h**) Pancreas. Nuclear immunoreactivity in necrotic acinar cells. Influenza A virus.

The following six chickens were subjected to histopathological analysis: two each from the groups inoculated with 10^6^ EID_50_ of Saga/23A2T and 10^5^ EID_50_ of Kagoshima/23B1T, and 10^3^ EID_50_ of Kagoshima/23B1T as controls. In the lung, there was diffuse pneumonia with numerous heterophilic and lymphohistiocytic infiltrations in the air capillaries, fibrinous exudation, and cellular debris. The interlobular septa were markedly dilated owing to edema (Figure 8a). In the spleen, multifocal to coalescing moderate necrosis of the ellipsoid and periellipsoidal white pulp (lymphocytolysis) with fibrin deposition was observed (Figure 8b). In the brain, multifocal small to large foci of necrosis were randomly observed in both the gray and white matter (Figure 8c). Adjacent to the necrotic foci, neuronal cell bodies were hypereosinophilic and shrunken with pyknotic nuclei (neuronal necrosis). In the pancreas, the acinar cells showed multifocal degeneration and necrosis (Figure 8d). In the liver, the sinusoids were dilated with congestion, and hepatocytes showed vacuolar degeneration and single-cell necrosis/apoptosis.

Immunohistochemically, influenza virus nucleoprotein antigens were detected mainly in the degenerative and necrotic areas corresponding to the histological findings (Figure 8e–h; Table S2), as well as in endothelial cells throughout the body and occasionally in inflammatory cells. Viral antigens were detected in the chickens inoculated with Kagoshima/23B1T (Figure S4). Additionally, viral antigen expression was detected in the cardiomyocytes, renal tubular epithelial cells, intestinal epithelium, lamina propria, and thymic epithelial cells in the medulla (Table S2); however, no obvious morphological changes were observed. Viral antigens are rarely observed in the lymphocytes of lymphoid tissues.

## 4. Discussion

In this study, we conducted a phylogenetic analysis of the full genomes of the H5N1- and H5N6-subtype HPAI viruses that caused all outbreaks in poultry premises and wild birds in Japan, and performed animal experiments to determine the infectivity and virulence of the representative isolates of both subtypes in Japan during the winter of 2023–2024.

**Figure 9:**
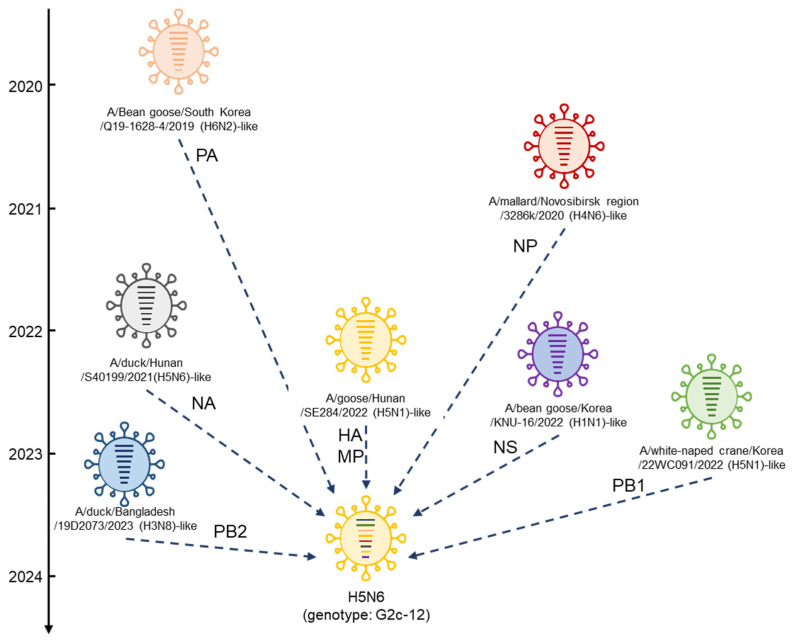
Formation of the index H5N6 G2c-12 genotype virus identified in Japan in the winter of 2023–2024. The eight bars indicate the eight segments (from top to bottom: PB2, PB1, PA, HA, NP, NA, M and NS). The colors of the individual bars of the H5N6 (genotype: G2c-12) virus indicate the closest donor virus isolate of the gene segment.

Two different genotypes, G2d-0 and G2c-12, were identified in Japan during the current season. The G2d-0 HPAI (H5N1) virus has been detected in Japan for three consecutive years, suggesting that this genotype may be better adapted to wild birds and maintained in wild bird populations. Notably, even within the G2d-0 genotype viruses, all eight segments of the H5N1 HPAI viruses isolated in the winter of 2023–2024 were genetically closer to those of the HPAI viruses isolated in the winter of 2021–2022 than to those isolated in the winter of 2022–2023. The G2d-0 genotype viruses isolated in the winter of 2022–2023 were genetically closer to the HPAI viruses isolated mainly in Alaska and on the west coast of North America during the winter of 2023–2024. HPAI outbreaks in poultry premises caused by the G2d-0 genotype virus have tended to occur mainly in northern Japan (e.g., Hokkaido, Aomori, and Iwate) during the past two seasons [11,22]. However, in the current season (winter of 2023–2024), outbreaks caused by the G2d-0 genotype virus were confirmed across a broad area, predominantly in the southern regions located below the Kanto region in southeastern and eastern areas of Japan, i.e., there was no HPAI outbreak on poultry premises in northern Japan during winter. We used the sequences uploaded to GISAID before 18 June 2024 to construct the phylogenetic tree, and to our knowledge, we identified the H5N1-subtype G2d-0 viruses derived from Korea for the first time (Figure 3) [23]. These results suggest that the G2d-0 genotype viruses in the current season spread to Japan via routes different from those of viruses in the previous two seasons. Although the HPAI virus was detected in wild birds in the northern regions of Japan (e.g., Hokkaido, Aomori, and Iwate), the change in migratory routes among the wild bird groups with G2d-0 in the 2022/2023 season may have resulted in a decrease in the concentration of the virus in the environment and prevented outbreaks in poultry in the northern areas. Among the G2c viruses isolated in Japan during the 2022/2023 season, eleven genotypes were reported; the viruses possessed multiple internal genes derived from wild bird AIVs [11]. An H5N6 virus isolated in the current season exhibited a new genotype designated as G2c-12; its NP and NS genes were closely related with those of H4N6 AIV isolated in Novosibirsk and the H1N1 AIV isolated in Korea, respectively. Moreover, the NA gene of the G2c-12 virus shared a common ancestor with H5N6 G2a subgroup HPAI viruses isolated in China. These results validate the previously reported suggestion that the genetic diversity of AIVs in Asia and Russia influences the emergence of numerous G2c genotypes [11]. Based on the phylogenetic analysis of the full genomes of the H5N6-subtype HPAI virus, this genotype may have emerged in East Asia and/or Siberia due to the reassortment of G2c subgroup HPAI viruses, G2a subtype HPAI viruses, and various avian influenza viruses (Figure 9). Similar to the G2d-0 genotype virus, all eight segments of the G2c-12 genotype virus phylogenetically formed a single cluster with the H5N6 HPAI virus isolated in Korea during the winter season. Collectively, it is reasonable that both genotypes of viruses isolated during the winter of 2023–2024 would intrude into Japan from Central and/or East Asia via migratory wild birds.

In animal experiments, the infectivity of representative isolates of both H5N1 and H5N6 subtypes was assessed based on virus shedding and the CLD_50_ value. No surviving chickens exhibited detectable antibodies against the influenza A virus or signs of viral shedding. This suggests that the CLD_50_ is aligned with the chicken infectious dose. The virulence of the isolates was evaluated based on MDT and pathological findings. The levels of infectivity of Saga/23A2T(H5N1) and Kagoshima/23B1T(H5N6) were equivalent. In addition, no change in chicken lethality was observed in Saga/23A2T compared to those with Hokkaido/22B3C, which belonged to the same group in the previous season. A previous report indicated that isolates belonging to the same subclade exhibit similar infectivity, and peak viral shedding is not significantly different among the subclades [24]. Notably, one chicken from the 10^6^ EID_50_ Kagoshima/23B1T inoculation group remained uninfected. Some H5-subtype isolates belonging to the clade 2.3.4.4b fail to infect all chickens, even at high doses, such as A/chicken/England/053052/2021, which is highly adapted to ducks, but less adapted to chickens [25]. The CLD_50_ of this isolate is 4.67 log_10_EID_50_, which is very close to that of Kagoshima/23B1T (4.60 log_10_EID_50_). Another study found that the adaptation of HPAI viruses to mallard ducks reduces their lethality in chickens, requiring high-dose inoculation to cause mortality. Even with a high-dose viral inoculation, a small number of chickens survive [26]. Notably, the CLD_50_ of H5N1 HAPIV isolated in 2004 (A/chicken/Yamaguchi/7/2004) was 2.0 log10 EID_50_ [27], indicating a 300-fold higher infectivity than that of the current isolates. One possibility is that the recent increase in CLD_50_ may be due to a reduction in adaptation to chickens over the past 20 years.

Our results suggest that the degrees of virulence of Saga/23A2T and Kagoshima/23B1T are indistinguishable. All chickens infected with both isolates died shortly after inoculation, exhibiting no obvious clinical signs, and presenting no lesions upon external examination. Although the MDT tended to be slightly longer for Saga/23A2T, this difference was not noteworthy when compared to our previous studies [9,10]. The most common pathological findings associated with H5-subtype HPAI viruses of clade 2.3.4.4b infection in poultry species are pancreatic and splenic necrosis [28,29]. However, in the present study, no necrotic foci or hemorrhages were macroscopically observed in these organs, and the main lesions consistently observed during necropsy were hydropericardium and pneumonia. Given that the reports of pancreatic and splenic necrosis and hemorrhages were based on post-mortem analyses of naturally affected birds, the inconsistent findings of the present study may be attributed to the acute conditions resulting from the high virus dose and short time to death.

Histopathologically, the most severely affected tissues were the lungs, spleen, brain, and pancreas. These lesions are consistent with those observed in recent studies on experimental infections with H5-subtype HPAI viruses of the 2.3.4.4b clade in chickens [30,31]. In clade 2.3.4.4, regardless of the subclades and NA subtypes, the major lesions, including parenchymal cell necrosis with lymphoplasmacytic and heterophilic inflammatory infiltrates, observed in affected chickens were similar, although there were some differences in the severity of lesions [24]. Immunohistochemistry demonstrated that the histological lesions observed in the systemic organs were caused by viral replication, suggesting that systemic infection and the associated multiple organ dysfunctions were the causes of mortality, as predicted. The number of chickens available for histological examination was limited; therefore, the reason for the variation in viral antigens among the isolates remains unclear. However, this may presumably be due to the different timings of autopsies after infection. In summary, the differences in isolates were unlikely to significantly influence tissue tropism or pathogenicity in the current study.

## 5. Conclusions

In this study, we characterized H5 HPAI isolates from Japanese domestic poultry during the 2023/2024 season, using a basic virological approach. We demonstrated that the common characteristics of HPAI viruses in previous experimental chicken infections, such as short incubation periods and high mortality rates, may not have changed. In addition, the outbreak periods were generally unchanged from those of previous seasons. These results indicate that the most important way to reduce the number of outbreaks is to maintain high levels of biosecurity during the winter season when HPAI viruses are detected in environments containing migratory birds. However, new genotypes with various internal genes closely related to AIVs isolated in Siberia and other Asian regions were found in Japan over four consecutive seasons, suggesting that frequent gene reassortment between HPAI viruses and other AIVs continues to occur in wild bird populations, including migratory birds. Additionally, the G2d-0 genotype was found for three consecutive years, suggesting the possibility that this genotype may be stably maintained in wild bird populations. In the future, we plan to analyze the molecular mechanism of the G2d-0 genotype, determine the reason for its stability in wild bird populations, and conduct a detailed analysis through comparative studies of pathogenicity in wild birds.

## Figures and Tables

**Table 1:** Virological characteristics of H5-subtype HPAI viruses showing different genotypes isolated in the 2022/2023 and 2023/2024 seasons.

Isolate	Subtype	Genotype	Season	CLD_50_	MDT	Mean Peak Viral Titer (log_10_EID_50_/mL)	
				(log_10_EID_50_)	(Day)	Trachea	Cloaca
Hokkaido/22B3C	H5N1	G2d-0	2022/2023	4.50	2.2	5.6 ± 0.3	4.9 ± 0.3
Saga/23A2T	H5N1	G2d-0	2023/2024	4.50	2.7	5.6 ± 0.4	4.0 ± 0.2
Kagoshima/23B1T	H5N6	G2c-12	2023/2024	4.60	2.3	5.4 ± 0.4	4.7 ± 0.2

CLD, chicken lethal dose; EID, egg infectious dose; MDT, mean death time.

## Data Availability

The data supporting this study are available from the corresponding author upon request.

## Supplementary Materials

The following supporting information can be downloaded at: https://www.mdpi.com/article/10.3390/v16121956/s1, Figure S1: Section of detailed phylogenetic trees focusing on the G2d-0 genotype (a. PB2, b. PB1, c. PA, d. NP, e. N1, f. MP, and g. NS); Figure S2: Section of the extended phylogenetic trees focusing on the G2c-12 genotype (a. PB2, b. PB1, c. PA, d. MP); Figure S3: Gross pathology of chickens inoculated with 10^6^ EID_50_ of Saga/23A2T (a,b) and Kagoshima/23B1T (c,d); Figure S4: Histological and immunohistochemical findings in chickens inoculated 106 EID50 Kagoshima/23B1T. (a) Lung, respiratory lobules, Interstitial pneumonia. Hematoxylin and eosin (HE). (b) Spleen. Necrosis of ellipsoid with fibrinous exudation. HE. (c) Cerebrum. Focal necrosis of neurons and glial cells. HE. (d) Pancreas. Focal necrosis of acinar cells. HE. (e) Lung, respiratory lobules. Strong intranuclear immunohistochemical signals were found in air capillaries. Immunolabeling for Influenza A virus. (f) Spleen. There is positive nuclear and cytoplasmic staining of ellipsoidal cells. Immunolabeling for Influenza A virus. (g) Cerebrum. Nuclei and dendrites of neurons are positive for Influenza A virus. Immunolabeling for Influenza A virus. (h) Pancreas. Nuclear immunoreactivity in necrotic acinar cells. Influenza A virus. Table S1: Information on representative H5 HPAIV isolates used in this study; Table S2: Immunohistochemical scoring of the major organs of chickens inoculated with Saga/23A2T and Kagoshima/23B1T.
