# Supplementary material for: Phylogenetic and Pathogenic Analysis of H5N1 and H5N6 High Pathogenicity Avian Influenza Virus Isolated from Poultry Farms (Layer and Broiler Chickens) in Japan in the 2023/2024 Season
Source: Viruses. 2024 Dec 20;16(12):1956. doi: 10.3390/v16121956 (PMC11680161; doi:10.3390/v16121956)
Supplement: Supplementary file 1 [file viruses-16-01956-s001.zip › Suppl. Figure S1.pptx]

## Slide 1
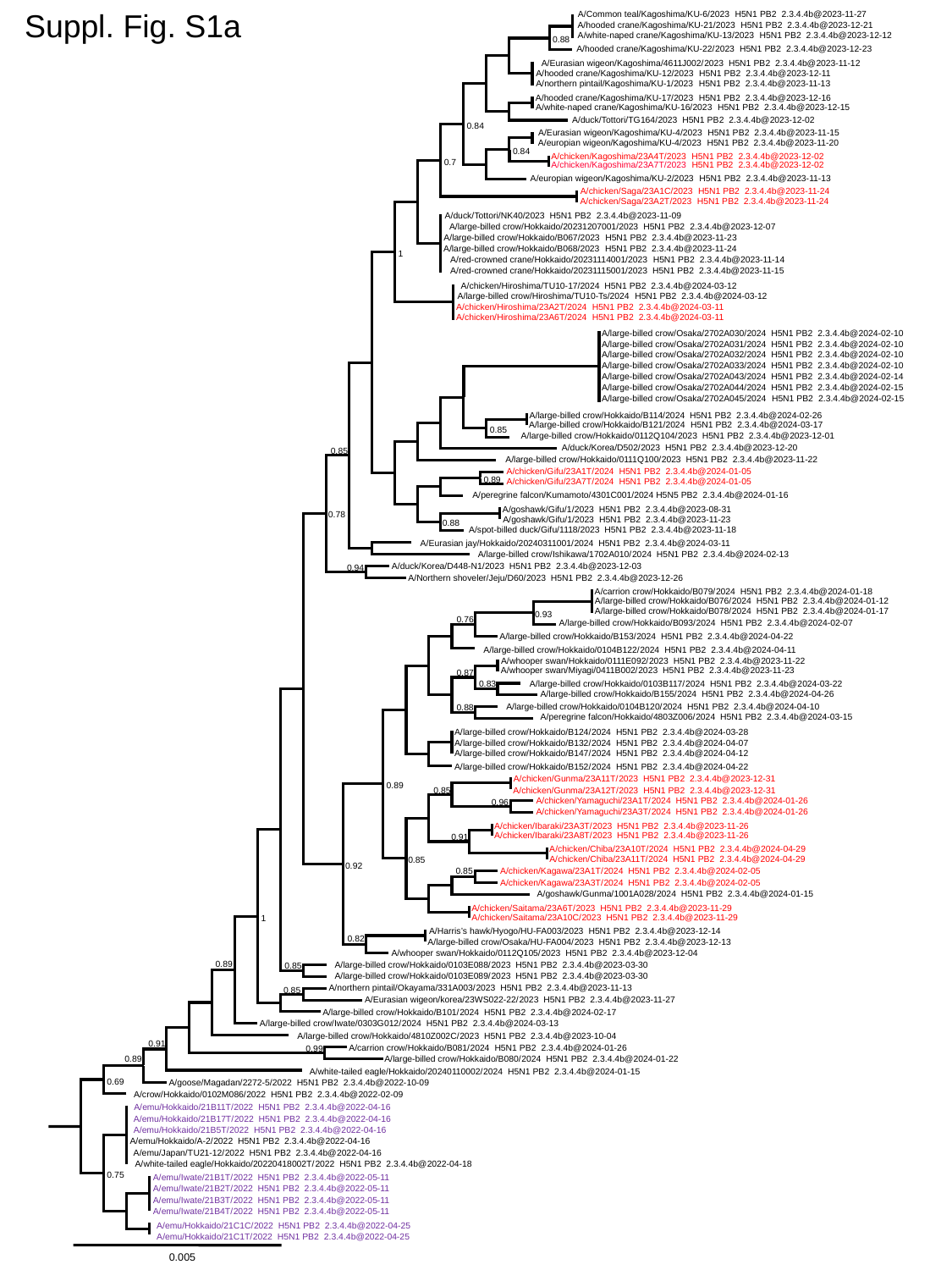

Suppl. Fig. S1a
A/Common teal/Kagoshima/KU-6/2023 H5N1 PB2 2.3.4.4b@2023-11-27
A/hooded crane/Kagoshima/KU-21/2023 H5N1 PB2 2.3.4.4b@2023-12-21
A/white-naped crane/Kagoshima/KU-13/2023 H5N1 PB2 2.3.4.4b@2023-12-12
0.88
A/hooded crane/Kagoshima/KU-22/2023 H5N1 PB2 2.3.4.4b@2023-12-23
A/Eurasian wigeon/Kagoshima/4611J002/2023 H5N1 PB2 2.3.4.4b@2023-11-12
A/hooded crane/Kagoshima/KU-12/2023 H5N1 PB2 2.3.4.4b@2023-12-11
A/northern pintail/Kagoshima/KU-1/2023 H5N1 PB2 2.3.4.4b@2023-11-13
A/hooded crane/Kagoshima/KU-17/2023 H5N1 PB2 2.3.4.4b@2023-12-16
A/white-naped crane/Kagoshima/KU-16/2023 H5N1 PB2 2.3.4.4b@2023-12-15
A/duck/Tottori/TG164/2023 H5N1 PB2 2.3.4.4b@2023-12-02
0.84
A/Eurasian wigeon/Kagoshima/KU-4/2023 H5N1 PB2 2.3.4.4b@2023-11-15
A/europian wigeon/Kagoshima/KU-4/2023 H5N1 PB2 2.3.4.4b@2023-11-20
0.84
A/chicken/Kagoshima/23A4T/2023 H5N1 PB2 2.3.4.4b@2023-12-02
0.7
A/chicken/Kagoshima/23A7T/2023 H5N1 PB2 2.3.4.4b@2023-12-02
A/europian wigeon/Kagoshima/KU-2/2023 H5N1 PB2 2.3.4.4b@2023-11-13
A/chicken/Saga/23A1C/2023 H5N1 PB2 2.3.4.4b@2023-11-24
A/chicken/Saga/23A2T/2023 H5N1 PB2 2.3.4.4b@2023-11-24
A/duck/Tottori/NK40/2023 H5N1 PB2 2.3.4.4b@2023-11-09
A/large-billed crow/Hokkaido/20231207001/2023 H5N1 PB2 2.3.4.4b@2023-12-07
A/large-billed crow/Hokkaido/B067/2023 H5N1 PB2 2.3.4.4b@2023-11-23
A/large-billed crow/Hokkaido/B068/2023 H5N1 PB2 2.3.4.4b@2023-11-24
1
A/red-crowned crane/Hokkaido/20231114001/2023 H5N1 PB2 2.3.4.4b@2023-11-14
A/red-crowned crane/Hokkaido/20231115001/2023 H5N1 PB2 2.3.4.4b@2023-11-15
A/chicken/Hiroshima/TU10-17/2024 H5N1 PB2 2.3.4.4b@2024-03-12
A/large-billed crow/Hiroshima/TU10-Ts/2024 H5N1 PB2 2.3.4.4b@2024-03-12
A/chicken/Hiroshima/23A2T/2024 H5N1 PB2 2.3.4.4b@2024-03-11
A/chicken/Hiroshima/23A6T/2024 H5N1 PB2 2.3.4.4b@2024-03-11
A/large-billed crow/Osaka/2702A030/2024 H5N1 PB2 2.3.4.4b@2024-02-10
A/large-billed crow/Osaka/2702A031/2024 H5N1 PB2 2.3.4.4b@2024-02-10
A/large-billed crow/Osaka/2702A032/2024 H5N1 PB2 2.3.4.4b@2024-02-10
A/large-billed crow/Osaka/2702A033/2024 H5N1 PB2 2.3.4.4b@2024-02-10
A/large-billed crow/Osaka/2702A043/2024 H5N1 PB2 2.3.4.4b@2024-02-14
A/large-billed crow/Osaka/2702A044/2024 H5N1 PB2 2.3.4.4b@2024-02-15
A/large-billed crow/Osaka/2702A045/2024 H5N1 PB2 2.3.4.4b@2024-02-15
A/large-billed crow/Hokkaido/B114/2024 H5N1 PB2 2.3.4.4b@2024-02-26
A/large-billed crow/Hokkaido/B121/2024 H5N1 PB2 2.3.4.4b@2024-03-17
0.85
A/large-billed crow/Hokkaido/0112Q104/2023 H5N1 PB2 2.3.4.4b@2023-12-01
A/duck/Korea/D502/2023 H5N1 PB2 2.3.4.4b@2023-12-20
0.85
A/large-billed crow/Hokkaido/0111Q100/2023 H5N1 PB2 2.3.4.4b@2023-11-22
A/chicken/Gifu/23A1T/2024 H5N1 PB2 2.3.4.4b@2024-01-05
0.89
A/chicken/Gifu/23A7T/2024 H5N1 PB2 2.3.4.4b@2024-01-05
A/peregrine falcon/Kumamoto/4301C001/2024 H5N5 PB2 2.3.4.4b@2024-01-16
A/goshawk/Gifu/1/2023 H5N1 PB2 2.3.4.4b@2023-08-31
0.78
A/goshawk/Gifu/1/2023 H5N1 PB2 2.3.4.4b@2023-11-23
0.88
A/spot-billed duck/Gifu/1118/2023 H5N1 PB2 2.3.4.4b@2023-11-18
A/Eurasian jay/Hokkaido/20240311001/2024 H5N1 PB2 2.3.4.4b@2024-03-11
A/large-billed crow/Ishikawa/1702A010/2024 H5N1 PB2 2.3.4.4b@2024-02-13
A/duck/Korea/D448-N1/2023 H5N1 PB2 2.3.4.4b@2023-12-03
0.94
A/Northern shoveler/Jeju/D60/2023 H5N1 PB2 2.3.4.4b@2023-12-26
A/carrion crow/Hokkaido/B079/2024 H5N1 PB2 2.3.4.4b@2024-01-18
A/large-billed crow/Hokkaido/B076/2024 H5N1 PB2 2.3.4.4b@2024-01-12
A/large-billed crow/Hokkaido/B078/2024 H5N1 PB2 2.3.4.4b@2024-01-17
0.93
0.76
A/large-billed crow/Hokkaido/B093/2024 H5N1 PB2 2.3.4.4b@2024-02-07
A/large-billed crow/Hokkaido/B153/2024 H5N1 PB2 2.3.4.4b@2024-04-22
A/large-billed crow/Hokkaido/0104B122/2024 H5N1 PB2 2.3.4.4b@2024-04-11
A/whooper swan/Hokkaido/0111E092/2023 H5N1 PB2 2.3.4.4b@2023-11-22
A/whooper swan/Miyagi/0411B002/2023 H5N1 PB2 2.3.4.4b@2023-11-23
0.87
A/large-billed crow/Hokkaido/0103B117/2024 H5N1 PB2 2.3.4.4b@2024-03-22
0.83
A/large-billed crow/Hokkaido/B155/2024 H5N1 PB2 2.3.4.4b@2024-04-26
A/large-billed crow/Hokkaido/0104B120/2024 H5N1 PB2 2.3.4.4b@2024-04-10
0.88
A/peregrine falcon/Hokkaido/4803Z006/2024 H5N1 PB2 2.3.4.4b@2024-03-15
A/large-billed crow/Hokkaido/B124/2024 H5N1 PB2 2.3.4.4b@2024-03-28
A/large-billed crow/Hokkaido/B132/2024 H5N1 PB2 2.3.4.4b@2024-04-07
A/large-billed crow/Hokkaido/B147/2024 H5N1 PB2 2.3.4.4b@2024-04-12
A/large-billed crow/Hokkaido/B152/2024 H5N1 PB2 2.3.4.4b@2024-04-22
A/chicken/Gunma/23A11T/2023 H5N1 PB2 2.3.4.4b@2023-12-31
0.89
0.85
A/chicken/Gunma/23A12T/2023 H5N1 PB2 2.3.4.4b@2023-12-31
A/chicken/Yamaguchi/23A1T/2024 H5N1 PB2 2.3.4.4b@2024-01-26
0.96
A/chicken/Yamaguchi/23A3T/2024 H5N1 PB2 2.3.4.4b@2024-01-26
A/chicken/Ibaraki/23A3T/2023 H5N1 PB2 2.3.4.4b@2023-11-26
A/chicken/Ibaraki/23A8T/2023 H5N1 PB2 2.3.4.4b@2023-11-26
0.91
A/chicken/Chiba/23A10T/2024 H5N1 PB2 2.3.4.4b@2024-04-29
A/chicken/Chiba/23A11T/2024 H5N1 PB2 2.3.4.4b@2024-04-29
0.85
0.92
0.85
A/chicken/Kagawa/23A1T/2024 H5N1 PB2 2.3.4.4b@2024-02-05
A/chicken/Kagawa/23A3T/2024 H5N1 PB2 2.3.4.4b@2024-02-05
A/goshawk/Gunma/1001A028/2024 H5N1 PB2 2.3.4.4b@2024-01-15
A/chicken/Saitama/23A6T/2023 H5N1 PB2 2.3.4.4b@2023-11-29
A/chicken/Saitama/23A10C/2023 H5N1 PB2 2.3.4.4b@2023-11-29
1
A/Harris’s hawk/Hyogo/HU-FA003/2023 H5N1 PB2 2.3.4.4b@2023-12-14
0.82
A/large-billed crow/Osaka/HU-FA004/2023 H5N1 PB2 2.3.4.4b@2023-12-13
A/whooper swan/Hokkaido/0112Q105/2023 H5N1 PB2 2.3.4.4b@2023-12-04
0.89
A/large-billed crow/Hokkaido/0103E088/2023 H5N1 PB2 2.3.4.4b@2023-03-30
0.85
A/large-billed crow/Hokkaido/0103E089/2023 H5N1 PB2 2.3.4.4b@2023-03-30
A/northern pintail/Okayama/331A003/2023 H5N1 PB2 2.3.4.4b@2023-11-13
0.85
A/Eurasian wigeon/korea/23WS022-22/2023 H5N1 PB2 2.3.4.4b@2023-11-27
A/large-billed crow/Hokkaido/B101/2024 H5N1 PB2 2.3.4.4b@2024-02-17
A/large-billed crow/Iwate/0303G012/2024 H5N1 PB2 2.3.4.4b@2024-03-13
A/large-billed crow/Hokkaido/4810Z002C/2023 H5N1 PB2 2.3.4.4b@2023-10-04
0.91
A/carrion crow/Hokkaido/B081/2024 H5N1 PB2 2.3.4.4b@2024-01-26
0.99
A/large-billed crow/Hokkaido/B080/2024 H5N1 PB2 2.3.4.4b@2024-01-22
0.89
A/white-tailed eagle/Hokkaido/20240110002/2024 H5N1 PB2 2.3.4.4b@2024-01-15
0.69
A/goose/Magadan/2272-5/2022 H5N1 PB2 2.3.4.4b@2022-10-09
A/crow/Hokkaido/0102M086/2022 H5N1 PB2 2.3.4.4b@2022-02-09
A/emu/Hokkaido/21B11T/2022 H5N1 PB2 2.3.4.4b@2022-04-16
A/emu/Hokkaido/21B17T/2022 H5N1 PB2 2.3.4.4b@2022-04-16
A/emu/Hokkaido/21B5T/2022 H5N1 PB2 2.3.4.4b@2022-04-16
A/emu/Hokkaido/A-2/2022 H5N1 PB2 2.3.4.4b@2022-04-16
A/emu/Japan/TU21-12/2022 H5N1 PB2 2.3.4.4b@2022-04-16
A/white-tailed eagle/Hokkaido/20220418002T/2022 H5N1 PB2 2.3.4.4b@2022-04-18
0.75
A/emu/Iwate/21B1T/2022 H5N1 PB2 2.3.4.4b@2022-05-11
A/emu/Iwate/21B2T/2022 H5N1 PB2 2.3.4.4b@2022-05-11
A/emu/Iwate/21B3T/2022 H5N1 PB2 2.3.4.4b@2022-05-11
A/emu/Iwate/21B4T/2022 H5N1 PB2 2.3.4.4b@2022-05-11
A/emu/Hokkaido/21C1C/2022 H5N1 PB2 2.3.4.4b@2022-04-25
A/emu/Hokkaido/21C1T/2022 H5N1 PB2 2.3.4.4b@2022-04-25
0.005

## Slide 2
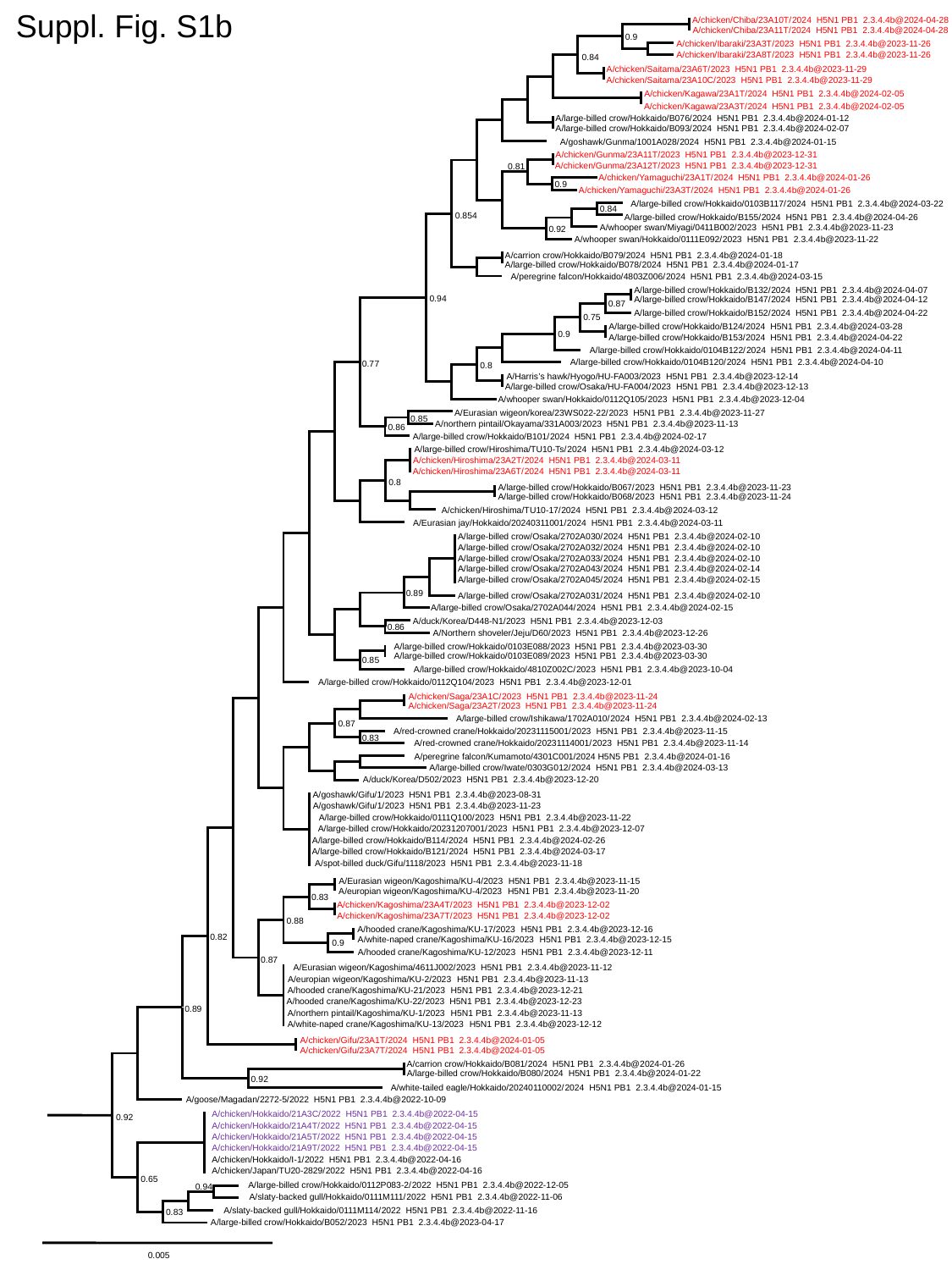

Suppl. Fig. S1b
A/chicken/Chiba/23A10T/2024 H5N1 PB1 2.3.4.4b@2024-04-28
A/chicken/Chiba/23A11T/2024 H5N1 PB1 2.3.4.4b@2024-04-28
0.9
A/chicken/Ibaraki/23A3T/2023 H5N1 PB1 2.3.4.4b@2023-11-26
A/chicken/Ibaraki/23A8T/2023 H5N1 PB1 2.3.4.4b@2023-11-26
0.84
A/chicken/Saitama/23A6T/2023 H5N1 PB1 2.3.4.4b@2023-11-29
A/chicken/Saitama/23A10C/2023 H5N1 PB1 2.3.4.4b@2023-11-29
A/chicken/Kagawa/23A1T/2024 H5N1 PB1 2.3.4.4b@2024-02-05
A/chicken/Kagawa/23A3T/2024 H5N1 PB1 2.3.4.4b@2024-02-05
A/large-billed crow/Hokkaido/B076/2024 H5N1 PB1 2.3.4.4b@2024-01-12
A/large-billed crow/Hokkaido/B093/2024 H5N1 PB1 2.3.4.4b@2024-02-07
A/goshawk/Gunma/1001A028/2024 H5N1 PB1 2.3.4.4b@2024-01-15
A/chicken/Gunma/23A11T/2023 H5N1 PB1 2.3.4.4b@2023-12-31
A/chicken/Gunma/23A12T/2023 H5N1 PB1 2.3.4.4b@2023-12-31
0.81
A/chicken/Yamaguchi/23A1T/2024 H5N1 PB1 2.3.4.4b@2024-01-26
0.9
A/chicken/Yamaguchi/23A3T/2024 H5N1 PB1 2.3.4.4b@2024-01-26
A/large-billed crow/Hokkaido/0103B117/2024 H5N1 PB1 2.3.4.4b@2024-03-22
0.84
0.854
A/large-billed crow/Hokkaido/B155/2024 H5N1 PB1 2.3.4.4b@2024-04-26
A/whooper swan/Miyagi/0411B002/2023 H5N1 PB1 2.3.4.4b@2023-11-23
0.92
A/whooper swan/Hokkaido/0111E092/2023 H5N1 PB1 2.3.4.4b@2023-11-22
A/carrion crow/Hokkaido/B079/2024 H5N1 PB1 2.3.4.4b@2024-01-18
A/large-billed crow/Hokkaido/B078/2024 H5N1 PB1 2.3.4.4b@2024-01-17
A/peregrine falcon/Hokkaido/4803Z006/2024 H5N1 PB1 2.3.4.4b@2024-03-15
A/large-billed crow/Hokkaido/B132/2024 H5N1 PB1 2.3.4.4b@2024-04-07
0.94
A/large-billed crow/Hokkaido/B147/2024 H5N1 PB1 2.3.4.4b@2024-04-12
0.87
A/large-billed crow/Hokkaido/B152/2024 H5N1 PB1 2.3.4.4b@2024-04-22
0.75
A/large-billed crow/Hokkaido/B124/2024 H5N1 PB1 2.3.4.4b@2024-03-28
0.9
A/large-billed crow/Hokkaido/B153/2024 H5N1 PB1 2.3.4.4b@2024-04-22
A/large-billed crow/Hokkaido/0104B122/2024 H5N1 PB1 2.3.4.4b@2024-04-11
A/large-billed crow/Hokkaido/0104B120/2024 H5N1 PB1 2.3.4.4b@2024-04-10
0.77
0.8
A/Harris’s hawk/Hyogo/HU-FA003/2023 H5N1 PB1 2.3.4.4b@2023-12-14
A/large-billed crow/Osaka/HU-FA004/2023 H5N1 PB1 2.3.4.4b@2023-12-13
A/whooper swan/Hokkaido/0112Q105/2023 H5N1 PB1 2.3.4.4b@2023-12-04
A/Eurasian wigeon/korea/23WS022-22/2023 H5N1 PB1 2.3.4.4b@2023-11-27
0.85
A/northern pintail/Okayama/331A003/2023 H5N1 PB1 2.3.4.4b@2023-11-13
0.86
A/large-billed crow/Hokkaido/B101/2024 H5N1 PB1 2.3.4.4b@2024-02-17
A/large-billed crow/Hiroshima/TU10-Ts/2024 H5N1 PB1 2.3.4.4b@2024-03-12
A/chicken/Hiroshima/23A2T/2024 H5N1 PB1 2.3.4.4b@2024-03-11
A/chicken/Hiroshima/23A6T/2024 H5N1 PB1 2.3.4.4b@2024-03-11
0.8
A/large-billed crow/Hokkaido/B067/2023 H5N1 PB1 2.3.4.4b@2023-11-23
A/large-billed crow/Hokkaido/B068/2023 H5N1 PB1 2.3.4.4b@2023-11-24
A/chicken/Hiroshima/TU10-17/2024 H5N1 PB1 2.3.4.4b@2024-03-12
A/Eurasian jay/Hokkaido/20240311001/2024 H5N1 PB1 2.3.4.4b@2024-03-11
A/large-billed crow/Osaka/2702A030/2024 H5N1 PB1 2.3.4.4b@2024-02-10
A/large-billed crow/Osaka/2702A032/2024 H5N1 PB1 2.3.4.4b@2024-02-10
A/large-billed crow/Osaka/2702A033/2024 H5N1 PB1 2.3.4.4b@2024-02-10
A/large-billed crow/Osaka/2702A043/2024 H5N1 PB1 2.3.4.4b@2024-02-14
A/large-billed crow/Osaka/2702A045/2024 H5N1 PB1 2.3.4.4b@2024-02-15
0.89
A/large-billed crow/Osaka/2702A031/2024 H5N1 PB1 2.3.4.4b@2024-02-10
A/large-billed crow/Osaka/2702A044/2024 H5N1 PB1 2.3.4.4b@2024-02-15
A/duck/Korea/D448-N1/2023 H5N1 PB1 2.3.4.4b@2023-12-03
0.86
A/Northern shoveler/Jeju/D60/2023 H5N1 PB1 2.3.4.4b@2023-12-26
A/large-billed crow/Hokkaido/0103E088/2023 H5N1 PB1 2.3.4.4b@2023-03-30
A/large-billed crow/Hokkaido/0103E089/2023 H5N1 PB1 2.3.4.4b@2023-03-30
0.85
A/large-billed crow/Hokkaido/4810Z002C/2023 H5N1 PB1 2.3.4.4b@2023-10-04
A/large-billed crow/Hokkaido/0112Q104/2023 H5N1 PB1 2.3.4.4b@2023-12-01
A/chicken/Saga/23A1C/2023 H5N1 PB1 2.3.4.4b@2023-11-24
A/chicken/Saga/23A2T/2023 H5N1 PB1 2.3.4.4b@2023-11-24
A/large-billed crow/Ishikawa/1702A010/2024 H5N1 PB1 2.3.4.4b@2024-02-13
0.87
A/red-crowned crane/Hokkaido/20231115001/2023 H5N1 PB1 2.3.4.4b@2023-11-15
0.83
A/red-crowned crane/Hokkaido/20231114001/2023 H5N1 PB1 2.3.4.4b@2023-11-14
A/peregrine falcon/Kumamoto/4301C001/2024 H5N5 PB1 2.3.4.4b@2024-01-16
A/large-billed crow/Iwate/0303G012/2024 H5N1 PB1 2.3.4.4b@2024-03-13
A/duck/Korea/D502/2023 H5N1 PB1 2.3.4.4b@2023-12-20
A/goshawk/Gifu/1/2023 H5N1 PB1 2.3.4.4b@2023-08-31
A/goshawk/Gifu/1/2023 H5N1 PB1 2.3.4.4b@2023-11-23
A/large-billed crow/Hokkaido/0111Q100/2023 H5N1 PB1 2.3.4.4b@2023-11-22
A/large-billed crow/Hokkaido/20231207001/2023 H5N1 PB1 2.3.4.4b@2023-12-07
A/large-billed crow/Hokkaido/B114/2024 H5N1 PB1 2.3.4.4b@2024-02-26
A/large-billed crow/Hokkaido/B121/2024 H5N1 PB1 2.3.4.4b@2024-03-17
A/spot-billed duck/Gifu/1118/2023 H5N1 PB1 2.3.4.4b@2023-11-18
A/Eurasian wigeon/Kagoshima/KU-4/2023 H5N1 PB1 2.3.4.4b@2023-11-15
A/europian wigeon/Kagoshima/KU-4/2023 H5N1 PB1 2.3.4.4b@2023-11-20
0.83
A/chicken/Kagoshima/23A4T/2023 H5N1 PB1 2.3.4.4b@2023-12-02
A/chicken/Kagoshima/23A7T/2023 H5N1 PB1 2.3.4.4b@2023-12-02
0.88
A/hooded crane/Kagoshima/KU-17/2023 H5N1 PB1 2.3.4.4b@2023-12-16
0.82
A/white-naped crane/Kagoshima/KU-16/2023 H5N1 PB1 2.3.4.4b@2023-12-15
0.9
A/hooded crane/Kagoshima/KU-12/2023 H5N1 PB1 2.3.4.4b@2023-12-11
0.87
A/Eurasian wigeon/Kagoshima/4611J002/2023 H5N1 PB1 2.3.4.4b@2023-11-12
A/europian wigeon/Kagoshima/KU-2/2023 H5N1 PB1 2.3.4.4b@2023-11-13
A/hooded crane/Kagoshima/KU-21/2023 H5N1 PB1 2.3.4.4b@2023-12-21
A/hooded crane/Kagoshima/KU-22/2023 H5N1 PB1 2.3.4.4b@2023-12-23
0.89
A/northern pintail/Kagoshima/KU-1/2023 H5N1 PB1 2.3.4.4b@2023-11-13
A/white-naped crane/Kagoshima/KU-13/2023 H5N1 PB1 2.3.4.4b@2023-12-12
A/chicken/Gifu/23A1T/2024 H5N1 PB1 2.3.4.4b@2024-01-05
A/chicken/Gifu/23A7T/2024 H5N1 PB1 2.3.4.4b@2024-01-05
A/carrion crow/Hokkaido/B081/2024 H5N1 PB1 2.3.4.4b@2024-01-26
A/large-billed crow/Hokkaido/B080/2024 H5N1 PB1 2.3.4.4b@2024-01-22
0.92
A/white-tailed eagle/Hokkaido/20240110002/2024 H5N1 PB1 2.3.4.4b@2024-01-15
A/goose/Magadan/2272-5/2022 H5N1 PB1 2.3.4.4b@2022-10-09
A/chicken/Hokkaido/21A3C/2022 H5N1 PB1 2.3.4.4b@2022-04-15
0.92
A/chicken/Hokkaido/21A4T/2022 H5N1 PB1 2.3.4.4b@2022-04-15
A/chicken/Hokkaido/21A5T/2022 H5N1 PB1 2.3.4.4b@2022-04-15
A/chicken/Hokkaido/21A9T/2022 H5N1 PB1 2.3.4.4b@2022-04-15
A/chicken/Hokkaido/I-1/2022 H5N1 PB1 2.3.4.4b@2022-04-16
A/chicken/Japan/TU20-2829/2022 H5N1 PB1 2.3.4.4b@2022-04-16
0.65
A/large-billed crow/Hokkaido/0112P083-2/2022 H5N1 PB1 2.3.4.4b@2022-12-05
0.94
A/slaty-backed gull/Hokkaido/0111M111/2022 H5N1 PB1 2.3.4.4b@2022-11-06
A/slaty-backed gull/Hokkaido/0111M114/2022 H5N1 PB1 2.3.4.4b@2022-11-16
0.83
A/large-billed crow/Hokkaido/B052/2023 H5N1 PB1 2.3.4.4b@2023-04-17
0.005

## Slide 3
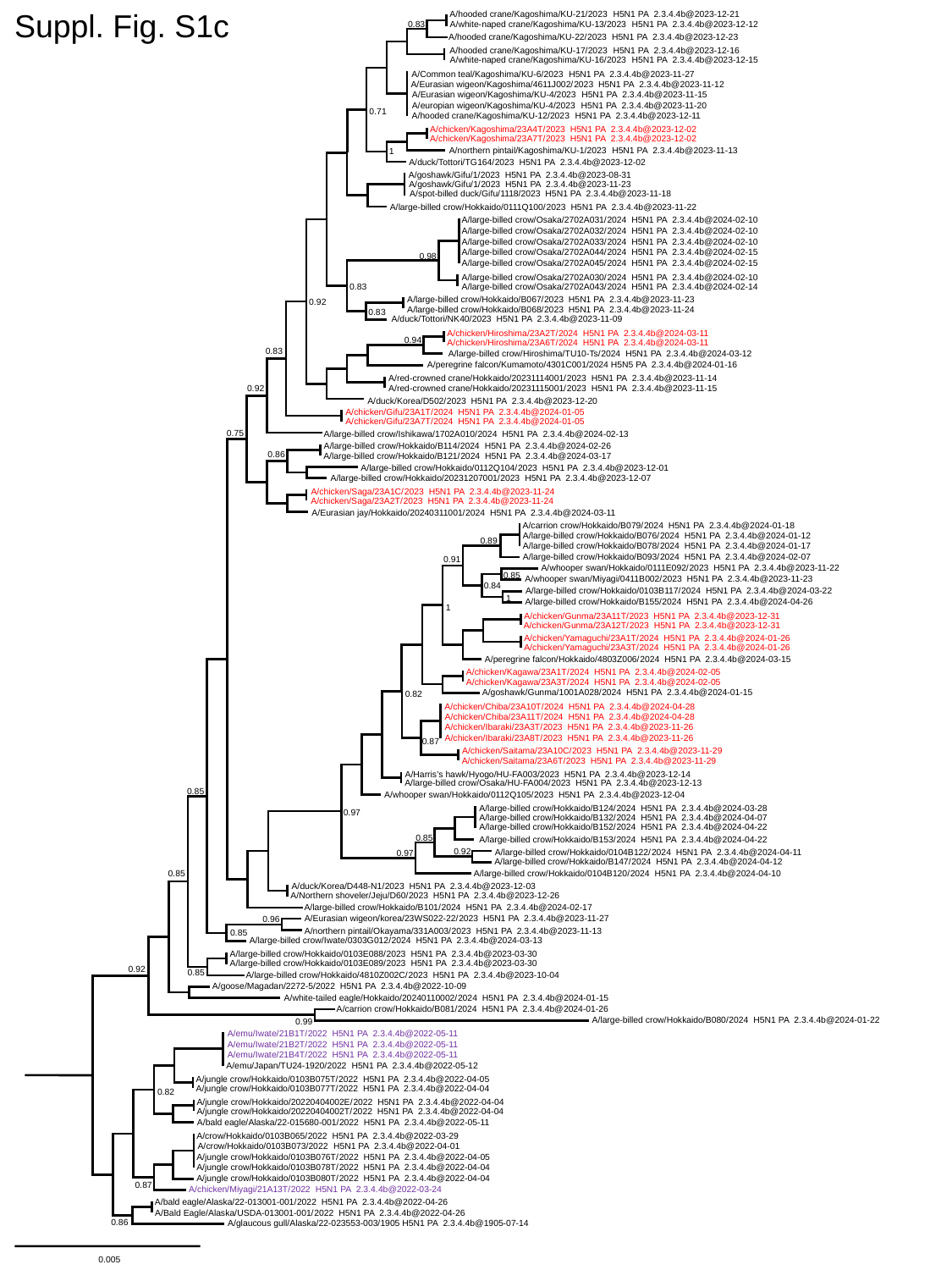

Suppl. Fig. S1c
A/hooded crane/Kagoshima/KU-21/2023 H5N1 PA 2.3.4.4b@2023-12-21
0.83
A/white-naped crane/Kagoshima/KU-13/2023 H5N1 PA 2.3.4.4b@2023-12-12
A/hooded crane/Kagoshima/KU-22/2023 H5N1 PA 2.3.4.4b@2023-12-23
A/hooded crane/Kagoshima/KU-17/2023 H5N1 PA 2.3.4.4b@2023-12-16
A/white-naped crane/Kagoshima/KU-16/2023 H5N1 PA 2.3.4.4b@2023-12-15
A/Common teal/Kagoshima/KU-6/2023 H5N1 PA 2.3.4.4b@2023-11-27
A/Eurasian wigeon/Kagoshima/4611J002/2023 H5N1 PA 2.3.4.4b@2023-11-12
A/Eurasian wigeon/Kagoshima/KU-4/2023 H5N1 PA 2.3.4.4b@2023-11-15
A/europian wigeon/Kagoshima/KU-4/2023 H5N1 PA 2.3.4.4b@2023-11-20
0.71
A/hooded crane/Kagoshima/KU-12/2023 H5N1 PA 2.3.4.4b@2023-12-11
A/chicken/Kagoshima/23A4T/2023 H5N1 PA 2.3.4.4b@2023-12-02
A/chicken/Kagoshima/23A7T/2023 H5N1 PA 2.3.4.4b@2023-12-02
A/northern pintail/Kagoshima/KU-1/2023 H5N1 PA 2.3.4.4b@2023-11-13
1
A/duck/Tottori/TG164/2023 H5N1 PA 2.3.4.4b@2023-12-02
A/goshawk/Gifu/1/2023 H5N1 PA 2.3.4.4b@2023-08-31
A/goshawk/Gifu/1/2023 H5N1 PA 2.3.4.4b@2023-11-23
A/spot-billed duck/Gifu/1118/2023 H5N1 PA 2.3.4.4b@2023-11-18
A/large-billed crow/Hokkaido/0111Q100/2023 H5N1 PA 2.3.4.4b@2023-11-22
A/large-billed crow/Osaka/2702A031/2024 H5N1 PA 2.3.4.4b@2024-02-10
A/large-billed crow/Osaka/2702A032/2024 H5N1 PA 2.3.4.4b@2024-02-10
A/large-billed crow/Osaka/2702A033/2024 H5N1 PA 2.3.4.4b@2024-02-10
A/large-billed crow/Osaka/2702A044/2024 H5N1 PA 2.3.4.4b@2024-02-15
0.98
A/large-billed crow/Osaka/2702A045/2024 H5N1 PA 2.3.4.4b@2024-02-15
A/large-billed crow/Osaka/2702A030/2024 H5N1 PA 2.3.4.4b@2024-02-10
A/large-billed crow/Osaka/2702A043/2024 H5N1 PA 2.3.4.4b@2024-02-14
0.83
A/large-billed crow/Hokkaido/B067/2023 H5N1 PA 2.3.4.4b@2023-11-23
0.92
A/large-billed crow/Hokkaido/B068/2023 H5N1 PA 2.3.4.4b@2023-11-24
0.83
A/duck/Tottori/NK40/2023 H5N1 PA 2.3.4.4b@2023-11-09
A/chicken/Hiroshima/23A2T/2024 H5N1 PA 2.3.4.4b@2024-03-11
0.94
A/chicken/Hiroshima/23A6T/2024 H5N1 PA 2.3.4.4b@2024-03-11
0.83
A/large-billed crow/Hiroshima/TU10-Ts/2024 H5N1 PA 2.3.4.4b@2024-03-12
A/peregrine falcon/Kumamoto/4301C001/2024 H5N5 PA 2.3.4.4b@2024-01-16
A/red-crowned crane/Hokkaido/20231114001/2023 H5N1 PA 2.3.4.4b@2023-11-14
0.92
A/red-crowned crane/Hokkaido/20231115001/2023 H5N1 PA 2.3.4.4b@2023-11-15
A/duck/Korea/D502/2023 H5N1 PA 2.3.4.4b@2023-12-20
A/chicken/Gifu/23A1T/2024 H5N1 PA 2.3.4.4b@2024-01-05
A/chicken/Gifu/23A7T/2024 H5N1 PA 2.3.4.4b@2024-01-05
0.75
A/large-billed crow/Ishikawa/1702A010/2024 H5N1 PA 2.3.4.4b@2024-02-13
A/large-billed crow/Hokkaido/B114/2024 H5N1 PA 2.3.4.4b@2024-02-26
0.86
A/large-billed crow/Hokkaido/B121/2024 H5N1 PA 2.3.4.4b@2024-03-17
A/large-billed crow/Hokkaido/0112Q104/2023 H5N1 PA 2.3.4.4b@2023-12-01
A/large-billed crow/Hokkaido/20231207001/2023 H5N1 PA 2.3.4.4b@2023-12-07
A/chicken/Saga/23A1C/2023 H5N1 PA 2.3.4.4b@2023-11-24
A/chicken/Saga/23A2T/2023 H5N1 PA 2.3.4.4b@2023-11-24
A/Eurasian jay/Hokkaido/20240311001/2024 H5N1 PA 2.3.4.4b@2024-03-11
A/carrion crow/Hokkaido/B079/2024 H5N1 PA 2.3.4.4b@2024-01-18
A/large-billed crow/Hokkaido/B076/2024 H5N1 PA 2.3.4.4b@2024-01-12
0.89
A/large-billed crow/Hokkaido/B078/2024 H5N1 PA 2.3.4.4b@2024-01-17
A/large-billed crow/Hokkaido/B093/2024 H5N1 PA 2.3.4.4b@2024-02-07
0.91
A/whooper swan/Hokkaido/0111E092/2023 H5N1 PA 2.3.4.4b@2023-11-22
0.85
A/whooper swan/Miyagi/0411B002/2023 H5N1 PA 2.3.4.4b@2023-11-23
0.84
A/large-billed crow/Hokkaido/0103B117/2024 H5N1 PA 2.3.4.4b@2024-03-22
1
A/large-billed crow/Hokkaido/B155/2024 H5N1 PA 2.3.4.4b@2024-04-26
1
A/chicken/Gunma/23A11T/2023 H5N1 PA 2.3.4.4b@2023-12-31
A/chicken/Gunma/23A12T/2023 H5N1 PA 2.3.4.4b@2023-12-31
A/chicken/Yamaguchi/23A1T/2024 H5N1 PA 2.3.4.4b@2024-01-26
A/chicken/Yamaguchi/23A3T/2024 H5N1 PA 2.3.4.4b@2024-01-26
A/peregrine falcon/Hokkaido/4803Z006/2024 H5N1 PA 2.3.4.4b@2024-03-15
A/chicken/Kagawa/23A1T/2024 H5N1 PA 2.3.4.4b@2024-02-05
A/chicken/Kagawa/23A3T/2024 H5N1 PA 2.3.4.4b@2024-02-05
A/goshawk/Gunma/1001A028/2024 H5N1 PA 2.3.4.4b@2024-01-15
0.82
A/chicken/Chiba/23A10T/2024 H5N1 PA 2.3.4.4b@2024-04-28
A/chicken/Chiba/23A11T/2024 H5N1 PA 2.3.4.4b@2024-04-28
A/chicken/Ibaraki/23A3T/2023 H5N1 PA 2.3.4.4b@2023-11-26
A/chicken/Ibaraki/23A8T/2023 H5N1 PA 2.3.4.4b@2023-11-26
0.87
A/chicken/Saitama/23A10C/2023 H5N1 PA 2.3.4.4b@2023-11-29
A/chicken/Saitama/23A6T/2023 H5N1 PA 2.3.4.4b@2023-11-29
A/Harris’s hawk/Hyogo/HU-FA003/2023 H5N1 PA 2.3.4.4b@2023-12-14
A/large-billed crow/Osaka/HU-FA004/2023 H5N1 PA 2.3.4.4b@2023-12-13
0.85
A/whooper swan/Hokkaido/0112Q105/2023 H5N1 PA 2.3.4.4b@2023-12-04
A/large-billed crow/Hokkaido/B124/2024 H5N1 PA 2.3.4.4b@2024-03-28
0.97
A/large-billed crow/Hokkaido/B132/2024 H5N1 PA 2.3.4.4b@2024-04-07
A/large-billed crow/Hokkaido/B152/2024 H5N1 PA 2.3.4.4b@2024-04-22
0.85
A/large-billed crow/Hokkaido/B153/2024 H5N1 PA 2.3.4.4b@2024-04-22
0.92
A/large-billed crow/Hokkaido/0104B122/2024 H5N1 PA 2.3.4.4b@2024-04-11
0.97
A/large-billed crow/Hokkaido/B147/2024 H5N1 PA 2.3.4.4b@2024-04-12
A/large-billed crow/Hokkaido/0104B120/2024 H5N1 PA 2.3.4.4b@2024-04-10
0.85
A/duck/Korea/D448-N1/2023 H5N1 PA 2.3.4.4b@2023-12-03
A/Northern shoveler/Jeju/D60/2023 H5N1 PA 2.3.4.4b@2023-12-26
A/large-billed crow/Hokkaido/B101/2024 H5N1 PA 2.3.4.4b@2024-02-17
A/Eurasian wigeon/korea/23WS022-22/2023 H5N1 PA 2.3.4.4b@2023-11-27
0.96
A/northern pintail/Okayama/331A003/2023 H5N1 PA 2.3.4.4b@2023-11-13
0.85
A/large-billed crow/Iwate/0303G012/2024 H5N1 PA 2.3.4.4b@2024-03-13
A/large-billed crow/Hokkaido/0103E088/2023 H5N1 PA 2.3.4.4b@2023-03-30
A/large-billed crow/Hokkaido/0103E089/2023 H5N1 PA 2.3.4.4b@2023-03-30
0.92
0.85
A/large-billed crow/Hokkaido/4810Z002C/2023 H5N1 PA 2.3.4.4b@2023-10-04
A/goose/Magadan/2272-5/2022 H5N1 PA 2.3.4.4b@2022-10-09
A/white-tailed eagle/Hokkaido/20240110002/2024 H5N1 PA 2.3.4.4b@2024-01-15
A/carrion crow/Hokkaido/B081/2024 H5N1 PA 2.3.4.4b@2024-01-26
A/large-billed crow/Hokkaido/B080/2024 H5N1 PA 2.3.4.4b@2024-01-22
0.99
A/emu/Iwate/21B1T/2022 H5N1 PA 2.3.4.4b@2022-05-11
A/emu/Iwate/21B2T/2022 H5N1 PA 2.3.4.4b@2022-05-11
A/emu/Iwate/21B4T/2022 H5N1 PA 2.3.4.4b@2022-05-11
A/emu/Japan/TU24-1920/2022 H5N1 PA 2.3.4.4b@2022-05-12
A/jungle crow/Hokkaido/0103B075T/2022 H5N1 PA 2.3.4.4b@2022-04-05
A/jungle crow/Hokkaido/0103B077T/2022 H5N1 PA 2.3.4.4b@2022-04-04
0.82
A/jungle crow/Hokkaido/20220404002E/2022 H5N1 PA 2.3.4.4b@2022-04-04
A/jungle crow/Hokkaido/20220404002T/2022 H5N1 PA 2.3.4.4b@2022-04-04
A/bald eagle/Alaska/22-015680-001/2022 H5N1 PA 2.3.4.4b@2022-05-11
A/crow/Hokkaido/0103B065/2022 H5N1 PA 2.3.4.4b@2022-03-29
A/crow/Hokkaido/0103B073/2022 H5N1 PA 2.3.4.4b@2022-04-01
A/jungle crow/Hokkaido/0103B076T/2022 H5N1 PA 2.3.4.4b@2022-04-05
A/jungle crow/Hokkaido/0103B078T/2022 H5N1 PA 2.3.4.4b@2022-04-04
A/jungle crow/Hokkaido/0103B080T/2022 H5N1 PA 2.3.4.4b@2022-04-04
0.87
A/chicken/Miyagi/21A13T/2022 H5N1 PA 2.3.4.4b@2022-03-24
A/bald eagle/Alaska/22-013001-001/2022 H5N1 PA 2.3.4.4b@2022-04-26
A/Bald Eagle/Alaska/USDA-013001-001/2022 H5N1 PA 2.3.4.4b@2022-04-26
0.86
A/glaucous gull/Alaska/22-023553-003/1905 H5N1 PA 2.3.4.4b@1905-07-14
0.005

## Slide 4
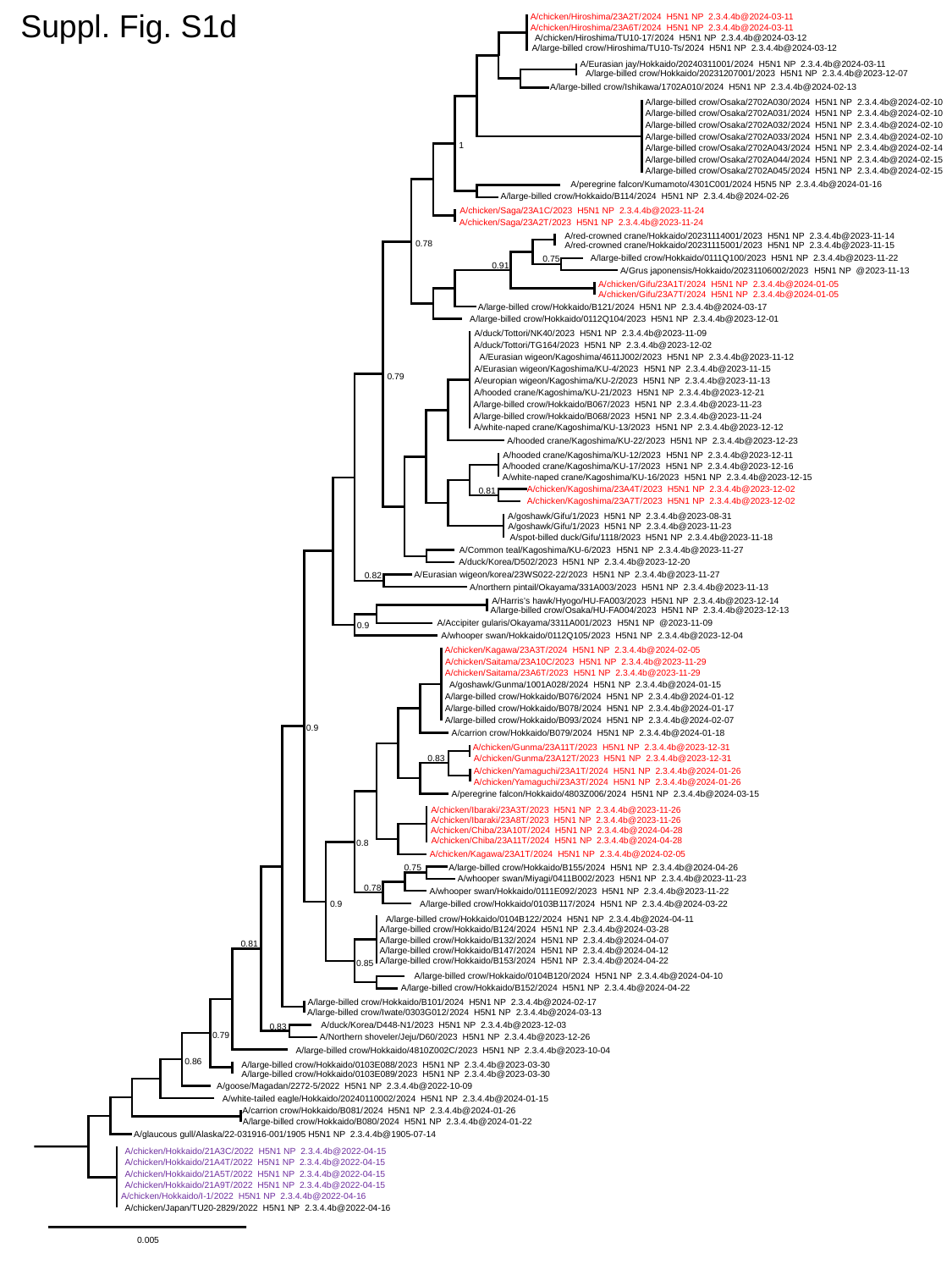

Suppl. Fig. S1d
A/chicken/Hiroshima/23A2T/2024 H5N1 NP 2.3.4.4b@2024-03-11
A/chicken/Hiroshima/23A6T/2024 H5N1 NP 2.3.4.4b@2024-03-11
A/chicken/Hiroshima/TU10-17/2024 H5N1 NP 2.3.4.4b@2024-03-12
A/large-billed crow/Hiroshima/TU10-Ts/2024 H5N1 NP 2.3.4.4b@2024-03-12
A/Eurasian jay/Hokkaido/20240311001/2024 H5N1 NP 2.3.4.4b@2024-03-11
A/large-billed crow/Hokkaido/20231207001/2023 H5N1 NP 2.3.4.4b@2023-12-07
A/large-billed crow/Ishikawa/1702A010/2024 H5N1 NP 2.3.4.4b@2024-02-13
A/large-billed crow/Osaka/2702A030/2024 H5N1 NP 2.3.4.4b@2024-02-10
A/large-billed crow/Osaka/2702A031/2024 H5N1 NP 2.3.4.4b@2024-02-10
A/large-billed crow/Osaka/2702A032/2024 H5N1 NP 2.3.4.4b@2024-02-10
A/large-billed crow/Osaka/2702A033/2024 H5N1 NP 2.3.4.4b@2024-02-10
1
A/large-billed crow/Osaka/2702A043/2024 H5N1 NP 2.3.4.4b@2024-02-14
A/large-billed crow/Osaka/2702A044/2024 H5N1 NP 2.3.4.4b@2024-02-15
A/large-billed crow/Osaka/2702A045/2024 H5N1 NP 2.3.4.4b@2024-02-15
A/peregrine falcon/Kumamoto/4301C001/2024 H5N5 NP 2.3.4.4b@2024-01-16
A/large-billed crow/Hokkaido/B114/2024 H5N1 NP 2.3.4.4b@2024-02-26
A/chicken/Saga/23A1C/2023 H5N1 NP 2.3.4.4b@2023-11-24
A/chicken/Saga/23A2T/2023 H5N1 NP 2.3.4.4b@2023-11-24
A/red-crowned crane/Hokkaido/20231114001/2023 H5N1 NP 2.3.4.4b@2023-11-14
0.78
A/red-crowned crane/Hokkaido/20231115001/2023 H5N1 NP 2.3.4.4b@2023-11-15
A/large-billed crow/Hokkaido/0111Q100/2023 H5N1 NP 2.3.4.4b@2023-11-22
0.75
0.91
A/Grus japonensis/Hokkaido/20231106002/2023 H5N1 NP @2023-11-13
A/chicken/Gifu/23A1T/2024 H5N1 NP 2.3.4.4b@2024-01-05
A/chicken/Gifu/23A7T/2024 H5N1 NP 2.3.4.4b@2024-01-05
A/large-billed crow/Hokkaido/B121/2024 H5N1 NP 2.3.4.4b@2024-03-17
A/large-billed crow/Hokkaido/0112Q104/2023 H5N1 NP 2.3.4.4b@2023-12-01
A/duck/Tottori/NK40/2023 H5N1 NP 2.3.4.4b@2023-11-09
A/duck/Tottori/TG164/2023 H5N1 NP 2.3.4.4b@2023-12-02
A/Eurasian wigeon/Kagoshima/4611J002/2023 H5N1 NP 2.3.4.4b@2023-11-12
A/Eurasian wigeon/Kagoshima/KU-4/2023 H5N1 NP 2.3.4.4b@2023-11-15
0.79
A/europian wigeon/Kagoshima/KU-2/2023 H5N1 NP 2.3.4.4b@2023-11-13
A/hooded crane/Kagoshima/KU-21/2023 H5N1 NP 2.3.4.4b@2023-12-21
A/large-billed crow/Hokkaido/B067/2023 H5N1 NP 2.3.4.4b@2023-11-23
A/large-billed crow/Hokkaido/B068/2023 H5N1 NP 2.3.4.4b@2023-11-24
A/white-naped crane/Kagoshima/KU-13/2023 H5N1 NP 2.3.4.4b@2023-12-12
A/hooded crane/Kagoshima/KU-22/2023 H5N1 NP 2.3.4.4b@2023-12-23
A/hooded crane/Kagoshima/KU-12/2023 H5N1 NP 2.3.4.4b@2023-12-11
A/hooded crane/Kagoshima/KU-17/2023 H5N1 NP 2.3.4.4b@2023-12-16
A/white-naped crane/Kagoshima/KU-16/2023 H5N1 NP 2.3.4.4b@2023-12-15
A/chicken/Kagoshima/23A4T/2023 H5N1 NP 2.3.4.4b@2023-12-02
0.81
A/chicken/Kagoshima/23A7T/2023 H5N1 NP 2.3.4.4b@2023-12-02
A/goshawk/Gifu/1/2023 H5N1 NP 2.3.4.4b@2023-08-31
A/goshawk/Gifu/1/2023 H5N1 NP 2.3.4.4b@2023-11-23
A/spot-billed duck/Gifu/1118/2023 H5N1 NP 2.3.4.4b@2023-11-18
A/Common teal/Kagoshima/KU-6/2023 H5N1 NP 2.3.4.4b@2023-11-27
A/duck/Korea/D502/2023 H5N1 NP 2.3.4.4b@2023-12-20
A/Eurasian wigeon/korea/23WS022-22/2023 H5N1 NP 2.3.4.4b@2023-11-27
0.82
A/northern pintail/Okayama/331A003/2023 H5N1 NP 2.3.4.4b@2023-11-13
A/Harris’s hawk/Hyogo/HU-FA003/2023 H5N1 NP 2.3.4.4b@2023-12-14
A/large-billed crow/Osaka/HU-FA004/2023 H5N1 NP 2.3.4.4b@2023-12-13
A/Accipiter gularis/Okayama/3311A001/2023 H5N1 NP @2023-11-09
0.9
A/whooper swan/Hokkaido/0112Q105/2023 H5N1 NP 2.3.4.4b@2023-12-04
A/chicken/Kagawa/23A3T/2024 H5N1 NP 2.3.4.4b@2024-02-05
A/chicken/Saitama/23A10C/2023 H5N1 NP 2.3.4.4b@2023-11-29
A/chicken/Saitama/23A6T/2023 H5N1 NP 2.3.4.4b@2023-11-29
A/goshawk/Gunma/1001A028/2024 H5N1 NP 2.3.4.4b@2024-01-15
A/large-billed crow/Hokkaido/B076/2024 H5N1 NP 2.3.4.4b@2024-01-12
A/large-billed crow/Hokkaido/B078/2024 H5N1 NP 2.3.4.4b@2024-01-17
A/large-billed crow/Hokkaido/B093/2024 H5N1 NP 2.3.4.4b@2024-02-07
0.9
A/carrion crow/Hokkaido/B079/2024 H5N1 NP 2.3.4.4b@2024-01-18
A/chicken/Gunma/23A11T/2023 H5N1 NP 2.3.4.4b@2023-12-31
A/chicken/Gunma/23A12T/2023 H5N1 NP 2.3.4.4b@2023-12-31
0.83
A/chicken/Yamaguchi/23A1T/2024 H5N1 NP 2.3.4.4b@2024-01-26
A/chicken/Yamaguchi/23A3T/2024 H5N1 NP 2.3.4.4b@2024-01-26
A/peregrine falcon/Hokkaido/4803Z006/2024 H5N1 NP 2.3.4.4b@2024-03-15
A/chicken/Ibaraki/23A3T/2023 H5N1 NP 2.3.4.4b@2023-11-26
A/chicken/Ibaraki/23A8T/2023 H5N1 NP 2.3.4.4b@2023-11-26
A/chicken/Chiba/23A10T/2024 H5N1 NP 2.3.4.4b@2024-04-28
A/chicken/Chiba/23A11T/2024 H5N1 NP 2.3.4.4b@2024-04-28
0.8
A/chicken/Kagawa/23A1T/2024 H5N1 NP 2.3.4.4b@2024-02-05
0.75
A/large-billed crow/Hokkaido/B155/2024 H5N1 NP 2.3.4.4b@2024-04-26
A/whooper swan/Miyagi/0411B002/2023 H5N1 NP 2.3.4.4b@2023-11-23
0.78
A/whooper swan/Hokkaido/0111E092/2023 H5N1 NP 2.3.4.4b@2023-11-22
0.9
A/large-billed crow/Hokkaido/0103B117/2024 H5N1 NP 2.3.4.4b@2024-03-22
A/large-billed crow/Hokkaido/0104B122/2024 H5N1 NP 2.3.4.4b@2024-04-11
A/large-billed crow/Hokkaido/B124/2024 H5N1 NP 2.3.4.4b@2024-03-28
A/large-billed crow/Hokkaido/B132/2024 H5N1 NP 2.3.4.4b@2024-04-07
0.81
A/large-billed crow/Hokkaido/B147/2024 H5N1 NP 2.3.4.4b@2024-04-12
A/large-billed crow/Hokkaido/B153/2024 H5N1 NP 2.3.4.4b@2024-04-22
0.85
A/large-billed crow/Hokkaido/0104B120/2024 H5N1 NP 2.3.4.4b@2024-04-10
A/large-billed crow/Hokkaido/B152/2024 H5N1 NP 2.3.4.4b@2024-04-22
A/large-billed crow/Hokkaido/B101/2024 H5N1 NP 2.3.4.4b@2024-02-17
A/large-billed crow/Iwate/0303G012/2024 H5N1 NP 2.3.4.4b@2024-03-13
A/duck/Korea/D448-N1/2023 H5N1 NP 2.3.4.4b@2023-12-03
0.83
0.79
A/Northern shoveler/Jeju/D60/2023 H5N1 NP 2.3.4.4b@2023-12-26
A/large-billed crow/Hokkaido/4810Z002C/2023 H5N1 NP 2.3.4.4b@2023-10-04
0.86
A/large-billed crow/Hokkaido/0103E088/2023 H5N1 NP 2.3.4.4b@2023-03-30
A/large-billed crow/Hokkaido/0103E089/2023 H5N1 NP 2.3.4.4b@2023-03-30
A/goose/Magadan/2272-5/2022 H5N1 NP 2.3.4.4b@2022-10-09
A/white-tailed eagle/Hokkaido/20240110002/2024 H5N1 NP 2.3.4.4b@2024-01-15
A/carrion crow/Hokkaido/B081/2024 H5N1 NP 2.3.4.4b@2024-01-26
A/large-billed crow/Hokkaido/B080/2024 H5N1 NP 2.3.4.4b@2024-01-22
A/glaucous gull/Alaska/22-031916-001/1905 H5N1 NP 2.3.4.4b@1905-07-14
A/chicken/Hokkaido/21A3C/2022 H5N1 NP 2.3.4.4b@2022-04-15
A/chicken/Hokkaido/21A4T/2022 H5N1 NP 2.3.4.4b@2022-04-15
A/chicken/Hokkaido/21A5T/2022 H5N1 NP 2.3.4.4b@2022-04-15
A/chicken/Hokkaido/21A9T/2022 H5N1 NP 2.3.4.4b@2022-04-15
A/chicken/Hokkaido/I-1/2022 H5N1 NP 2.3.4.4b@2022-04-16
A/chicken/Japan/TU20-2829/2022 H5N1 NP 2.3.4.4b@2022-04-16
0.005

## Slide 5
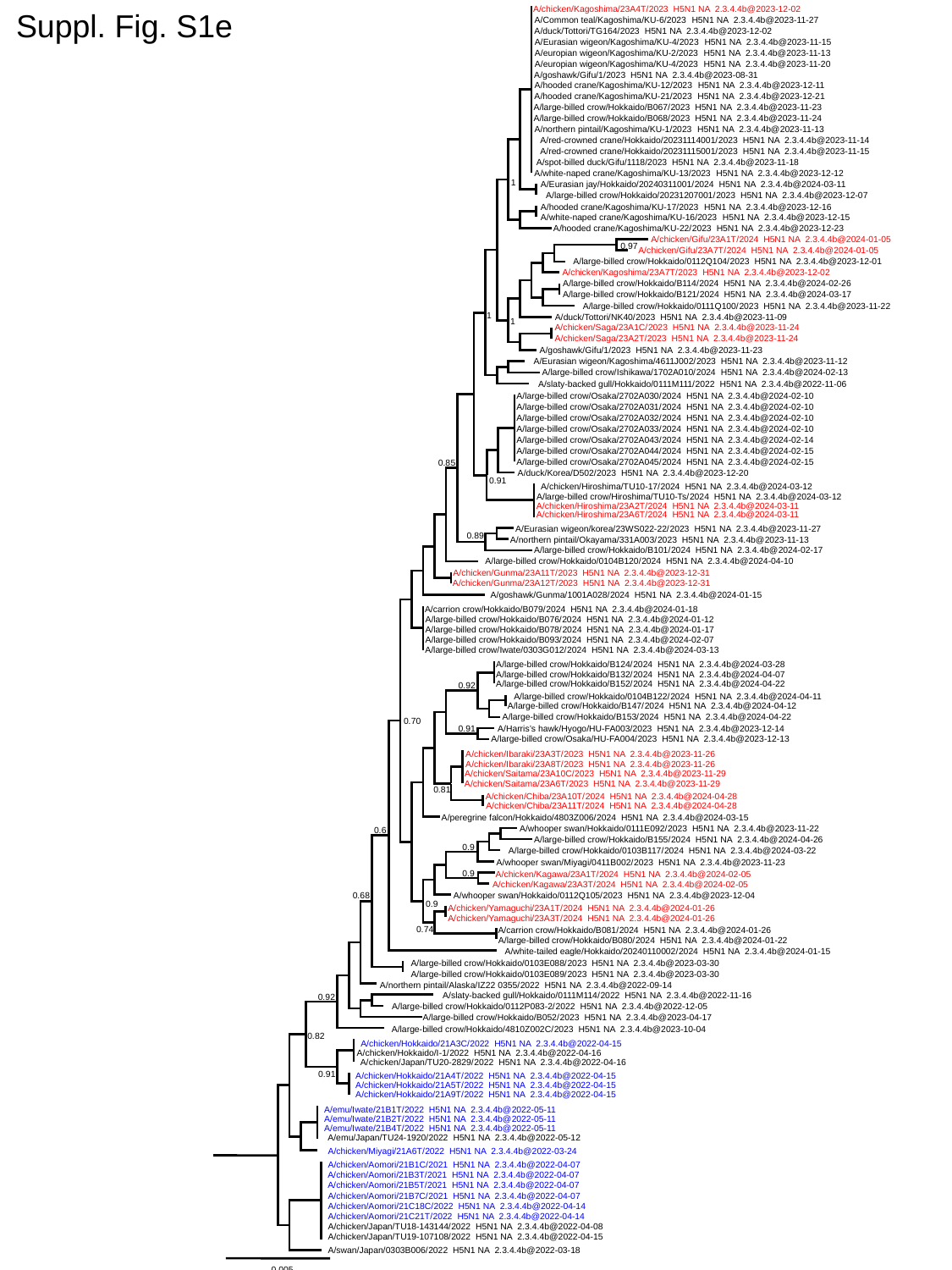

Suppl. Fig. S1e
A/chicken/Kagoshima/23A4T/2023 H5N1 NA 2.3.4.4b@2023-12-02
A/Common teal/Kagoshima/KU-6/2023 H5N1 NA 2.3.4.4b@2023-11-27
A/duck/Tottori/TG164/2023 H5N1 NA 2.3.4.4b@2023-12-02
A/Eurasian wigeon/Kagoshima/KU-4/2023 H5N1 NA 2.3.4.4b@2023-11-15
A/europian wigeon/Kagoshima/KU-2/2023 H5N1 NA 2.3.4.4b@2023-11-13
A/europian wigeon/Kagoshima/KU-4/2023 H5N1 NA 2.3.4.4b@2023-11-20
A/goshawk/Gifu/1/2023 H5N1 NA 2.3.4.4b@2023-08-31
A/hooded crane/Kagoshima/KU-12/2023 H5N1 NA 2.3.4.4b@2023-12-11
A/hooded crane/Kagoshima/KU-21/2023 H5N1 NA 2.3.4.4b@2023-12-21
A/large-billed crow/Hokkaido/B067/2023 H5N1 NA 2.3.4.4b@2023-11-23
A/large-billed crow/Hokkaido/B068/2023 H5N1 NA 2.3.4.4b@2023-11-24
A/northern pintail/Kagoshima/KU-1/2023 H5N1 NA 2.3.4.4b@2023-11-13
A/red-crowned crane/Hokkaido/20231114001/2023 H5N1 NA 2.3.4.4b@2023-11-14
A/red-crowned crane/Hokkaido/20231115001/2023 H5N1 NA 2.3.4.4b@2023-11-15
A/spot-billed duck/Gifu/1118/2023 H5N1 NA 2.3.4.4b@2023-11-18
A/white-naped crane/Kagoshima/KU-13/2023 H5N1 NA 2.3.4.4b@2023-12-12
1
A/Eurasian jay/Hokkaido/20240311001/2024 H5N1 NA 2.3.4.4b@2024-03-11
A/large-billed crow/Hokkaido/20231207001/2023 H5N1 NA 2.3.4.4b@2023-12-07
A/hooded crane/Kagoshima/KU-17/2023 H5N1 NA 2.3.4.4b@2023-12-16
A/white-naped crane/Kagoshima/KU-16/2023 H5N1 NA 2.3.4.4b@2023-12-15
A/hooded crane/Kagoshima/KU-22/2023 H5N1 NA 2.3.4.4b@2023-12-23
A/chicken/Gifu/23A1T/2024 H5N1 NA 2.3.4.4b@2024-01-05
0.97
A/chicken/Gifu/23A7T/2024 H5N1 NA 2.3.4.4b@2024-01-05
A/large-billed crow/Hokkaido/0112Q104/2023 H5N1 NA 2.3.4.4b@2023-12-01
A/chicken/Kagoshima/23A7T/2023 H5N1 NA 2.3.4.4b@2023-12-02
A/large-billed crow/Hokkaido/B114/2024 H5N1 NA 2.3.4.4b@2024-02-26
A/large-billed crow/Hokkaido/B121/2024 H5N1 NA 2.3.4.4b@2024-03-17
A/large-billed crow/Hokkaido/0111Q100/2023 H5N1 NA 2.3.4.4b@2023-11-22
1
A/duck/Tottori/NK40/2023 H5N1 NA 2.3.4.4b@2023-11-09
1
A/chicken/Saga/23A1C/2023 H5N1 NA 2.3.4.4b@2023-11-24
A/chicken/Saga/23A2T/2023 H5N1 NA 2.3.4.4b@2023-11-24
A/goshawk/Gifu/1/2023 H5N1 NA 2.3.4.4b@2023-11-23
A/Eurasian wigeon/Kagoshima/4611J002/2023 H5N1 NA 2.3.4.4b@2023-11-12
A/large-billed crow/Ishikawa/1702A010/2024 H5N1 NA 2.3.4.4b@2024-02-13
A/slaty-backed gull/Hokkaido/0111M111/2022 H5N1 NA 2.3.4.4b@2022-11-06
A/large-billed crow/Osaka/2702A030/2024 H5N1 NA 2.3.4.4b@2024-02-10
A/large-billed crow/Osaka/2702A031/2024 H5N1 NA 2.3.4.4b@2024-02-10
A/large-billed crow/Osaka/2702A032/2024 H5N1 NA 2.3.4.4b@2024-02-10
A/large-billed crow/Osaka/2702A033/2024 H5N1 NA 2.3.4.4b@2024-02-10
A/large-billed crow/Osaka/2702A043/2024 H5N1 NA 2.3.4.4b@2024-02-14
A/large-billed crow/Osaka/2702A044/2024 H5N1 NA 2.3.4.4b@2024-02-15
A/large-billed crow/Osaka/2702A045/2024 H5N1 NA 2.3.4.4b@2024-02-15
0.85
A/duck/Korea/D502/2023 H5N1 NA 2.3.4.4b@2023-12-20
0.91
A/chicken/Hiroshima/TU10-17/2024 H5N1 NA 2.3.4.4b@2024-03-12
A/large-billed crow/Hiroshima/TU10-Ts/2024 H5N1 NA 2.3.4.4b@2024-03-12
A/chicken/Hiroshima/23A2T/2024 H5N1 NA 2.3.4.4b@2024-03-11
A/chicken/Hiroshima/23A6T/2024 H5N1 NA 2.3.4.4b@2024-03-11
A/Eurasian wigeon/korea/23WS022-22/2023 H5N1 NA 2.3.4.4b@2023-11-27
0.89
A/northern pintail/Okayama/331A003/2023 H5N1 NA 2.3.4.4b@2023-11-13
A/large-billed crow/Hokkaido/B101/2024 H5N1 NA 2.3.4.4b@2024-02-17
A/large-billed crow/Hokkaido/0104B120/2024 H5N1 NA 2.3.4.4b@2024-04-10
A/chicken/Gunma/23A11T/2023 H5N1 NA 2.3.4.4b@2023-12-31
A/chicken/Gunma/23A12T/2023 H5N1 NA 2.3.4.4b@2023-12-31
A/goshawk/Gunma/1001A028/2024 H5N1 NA 2.3.4.4b@2024-01-15
A/carrion crow/Hokkaido/B079/2024 H5N1 NA 2.3.4.4b@2024-01-18
A/large-billed crow/Hokkaido/B076/2024 H5N1 NA 2.3.4.4b@2024-01-12
A/large-billed crow/Hokkaido/B078/2024 H5N1 NA 2.3.4.4b@2024-01-17
A/large-billed crow/Hokkaido/B093/2024 H5N1 NA 2.3.4.4b@2024-02-07
A/large-billed crow/Iwate/0303G012/2024 H5N1 NA 2.3.4.4b@2024-03-13
A/large-billed crow/Hokkaido/B124/2024 H5N1 NA 2.3.4.4b@2024-03-28
A/large-billed crow/Hokkaido/B132/2024 H5N1 NA 2.3.4.4b@2024-04-07
A/large-billed crow/Hokkaido/B152/2024 H5N1 NA 2.3.4.4b@2024-04-22
0.92
A/large-billed crow/Hokkaido/0104B122/2024 H5N1 NA 2.3.4.4b@2024-04-11
A/large-billed crow/Hokkaido/B147/2024 H5N1 NA 2.3.4.4b@2024-04-12
A/large-billed crow/Hokkaido/B153/2024 H5N1 NA 2.3.4.4b@2024-04-22
0.70
0.91
A/Harris’s hawk/Hyogo/HU-FA003/2023 H5N1 NA 2.3.4.4b@2023-12-14
A/large-billed crow/Osaka/HU-FA004/2023 H5N1 NA 2.3.4.4b@2023-12-13
A/chicken/Ibaraki/23A3T/2023 H5N1 NA 2.3.4.4b@2023-11-26
A/chicken/Ibaraki/23A8T/2023 H5N1 NA 2.3.4.4b@2023-11-26
A/chicken/Saitama/23A10C/2023 H5N1 NA 2.3.4.4b@2023-11-29
A/chicken/Saitama/23A6T/2023 H5N1 NA 2.3.4.4b@2023-11-29
0.81
A/chicken/Chiba/23A10T/2024 H5N1 NA 2.3.4.4b@2024-04-28
A/chicken/Chiba/23A11T/2024 H5N1 NA 2.3.4.4b@2024-04-28
A/peregrine falcon/Hokkaido/4803Z006/2024 H5N1 NA 2.3.4.4b@2024-03-15
A/whooper swan/Hokkaido/0111E092/2023 H5N1 NA 2.3.4.4b@2023-11-22
0.6
A/large-billed crow/Hokkaido/B155/2024 H5N1 NA 2.3.4.4b@2024-04-26
0.9
A/large-billed crow/Hokkaido/0103B117/2024 H5N1 NA 2.3.4.4b@2024-03-22
A/whooper swan/Miyagi/0411B002/2023 H5N1 NA 2.3.4.4b@2023-11-23
0.9
A/chicken/Kagawa/23A1T/2024 H5N1 NA 2.3.4.4b@2024-02-05
A/chicken/Kagawa/23A3T/2024 H5N1 NA 2.3.4.4b@2024-02-05
A/whooper swan/Hokkaido/0112Q105/2023 H5N1 NA 2.3.4.4b@2023-12-04
0.68
0.9
A/chicken/Yamaguchi/23A1T/2024 H5N1 NA 2.3.4.4b@2024-01-26
A/chicken/Yamaguchi/23A3T/2024 H5N1 NA 2.3.4.4b@2024-01-26
0.74
A/carrion crow/Hokkaido/B081/2024 H5N1 NA 2.3.4.4b@2024-01-26
A/large-billed crow/Hokkaido/B080/2024 H5N1 NA 2.3.4.4b@2024-01-22
A/white-tailed eagle/Hokkaido/20240110002/2024 H5N1 NA 2.3.4.4b@2024-01-15
A/large-billed crow/Hokkaido/0103E088/2023 H5N1 NA 2.3.4.4b@2023-03-30
A/large-billed crow/Hokkaido/0103E089/2023 H5N1 NA 2.3.4.4b@2023-03-30
A/northern pintail/Alaska/IZ22 0355/2022 H5N1 NA 2.3.4.4b@2022-09-14
A/slaty-backed gull/Hokkaido/0111M114/2022 H5N1 NA 2.3.4.4b@2022-11-16
0.92
A/large-billed crow/Hokkaido/0112P083-2/2022 H5N1 NA 2.3.4.4b@2022-12-05
A/large-billed crow/Hokkaido/B052/2023 H5N1 NA 2.3.4.4b@2023-04-17
A/large-billed crow/Hokkaido/4810Z002C/2023 H5N1 NA 2.3.4.4b@2023-10-04
0.82
A/chicken/Hokkaido/21A3C/2022 H5N1 NA 2.3.4.4b@2022-04-15
A/chicken/Hokkaido/I-1/2022 H5N1 NA 2.3.4.4b@2022-04-16
A/chicken/Japan/TU20-2829/2022 H5N1 NA 2.3.4.4b@2022-04-16
0.91
A/chicken/Hokkaido/21A4T/2022 H5N1 NA 2.3.4.4b@2022-04-15
A/chicken/Hokkaido/21A5T/2022 H5N1 NA 2.3.4.4b@2022-04-15
A/chicken/Hokkaido/21A9T/2022 H5N1 NA 2.3.4.4b@2022-04-15
A/emu/Iwate/21B1T/2022 H5N1 NA 2.3.4.4b@2022-05-11
A/emu/Iwate/21B2T/2022 H5N1 NA 2.3.4.4b@2022-05-11
A/emu/Iwate/21B4T/2022 H5N1 NA 2.3.4.4b@2022-05-11
A/emu/Japan/TU24-1920/2022 H5N1 NA 2.3.4.4b@2022-05-12
A/chicken/Miyagi/21A6T/2022 H5N1 NA 2.3.4.4b@2022-03-24
A/chicken/Aomori/21B1C/2021 H5N1 NA 2.3.4.4b@2022-04-07
A/chicken/Aomori/21B3T/2021 H5N1 NA 2.3.4.4b@2022-04-07
A/chicken/Aomori/21B5T/2021 H5N1 NA 2.3.4.4b@2022-04-07
A/chicken/Aomori/21B7C/2021 H5N1 NA 2.3.4.4b@2022-04-07
A/chicken/Aomori/21C18C/2022 H5N1 NA 2.3.4.4b@2022-04-14
A/chicken/Aomori/21C21T/2022 H5N1 NA 2.3.4.4b@2022-04-14
A/chicken/Japan/TU18-143144/2022 H5N1 NA 2.3.4.4b@2022-04-08
A/chicken/Japan/TU19-107108/2022 H5N1 NA 2.3.4.4b@2022-04-15
A/swan/Japan/0303B006/2022 H5N1 NA 2.3.4.4b@2022-03-18
0.005

## Slide 6
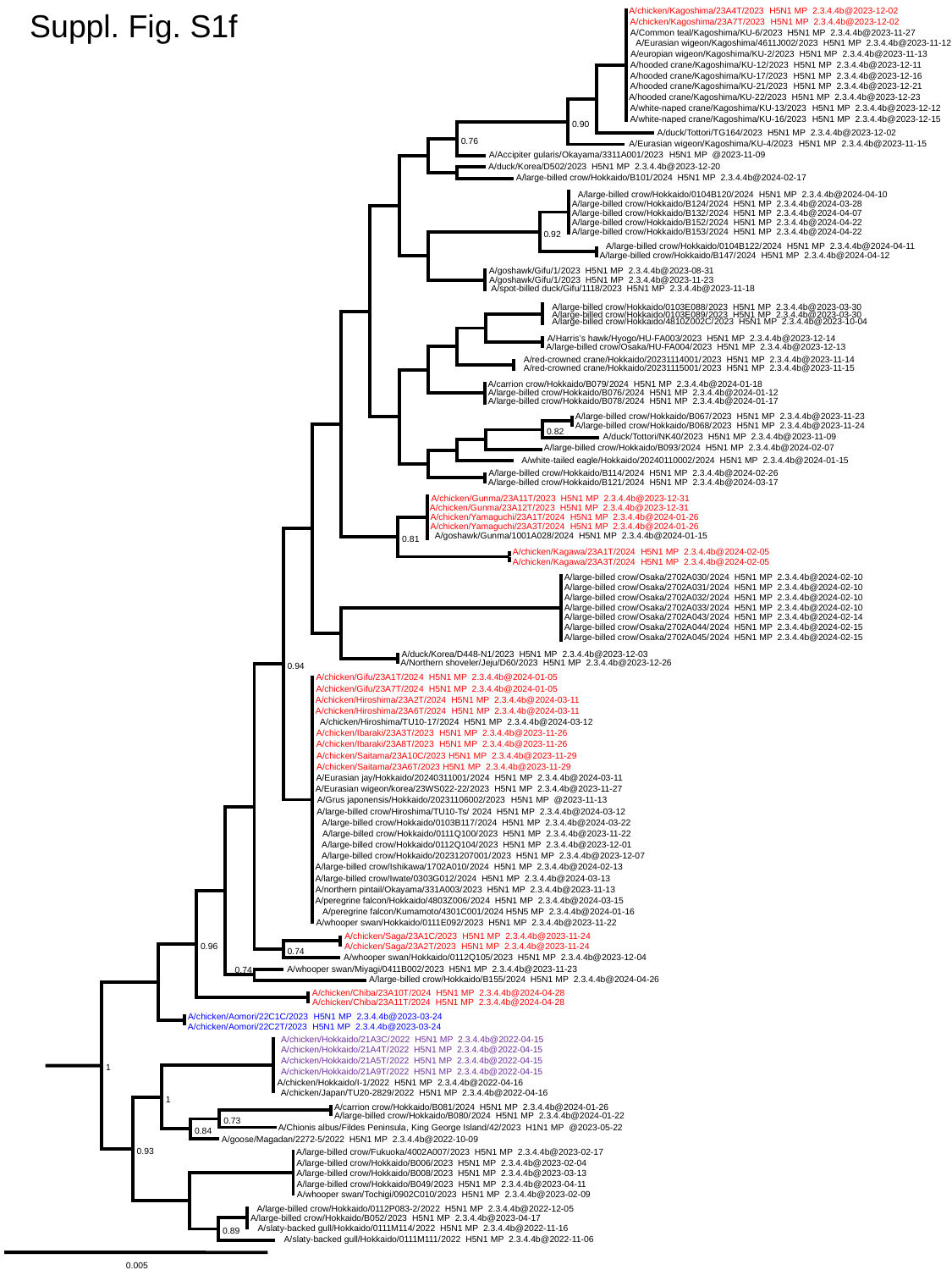

Suppl. Fig. S1f
A/chicken/Kagoshima/23A4T/2023 H5N1 MP 2.3.4.4b@2023-12-02
A/chicken/Kagoshima/23A7T/2023 H5N1 MP 2.3.4.4b@2023-12-02
A/Common teal/Kagoshima/KU-6/2023 H5N1 MP 2.3.4.4b@2023-11-27
A/Eurasian wigeon/Kagoshima/4611J002/2023 H5N1 MP 2.3.4.4b@2023-11-12
A/europian wigeon/Kagoshima/KU-2/2023 H5N1 MP 2.3.4.4b@2023-11-13
A/hooded crane/Kagoshima/KU-12/2023 H5N1 MP 2.3.4.4b@2023-12-11
A/hooded crane/Kagoshima/KU-17/2023 H5N1 MP 2.3.4.4b@2023-12-16
A/hooded crane/Kagoshima/KU-21/2023 H5N1 MP 2.3.4.4b@2023-12-21
A/hooded crane/Kagoshima/KU-22/2023 H5N1 MP 2.3.4.4b@2023-12-23
A/white-naped crane/Kagoshima/KU-13/2023 H5N1 MP 2.3.4.4b@2023-12-12
A/white-naped crane/Kagoshima/KU-16/2023 H5N1 MP 2.3.4.4b@2023-12-15
0.90
A/duck/Tottori/TG164/2023 H5N1 MP 2.3.4.4b@2023-12-02
0.76
A/Eurasian wigeon/Kagoshima/KU-4/2023 H5N1 MP 2.3.4.4b@2023-11-15
A/Accipiter gularis/Okayama/3311A001/2023 H5N1 MP @2023-11-09
A/duck/Korea/D502/2023 H5N1 MP 2.3.4.4b@2023-12-20
A/large-billed crow/Hokkaido/B101/2024 H5N1 MP 2.3.4.4b@2024-02-17
A/large-billed crow/Hokkaido/0104B120/2024 H5N1 MP 2.3.4.4b@2024-04-10
A/large-billed crow/Hokkaido/B124/2024 H5N1 MP 2.3.4.4b@2024-03-28
A/large-billed crow/Hokkaido/B132/2024 H5N1 MP 2.3.4.4b@2024-04-07
A/large-billed crow/Hokkaido/B152/2024 H5N1 MP 2.3.4.4b@2024-04-22
A/large-billed crow/Hokkaido/B153/2024 H5N1 MP 2.3.4.4b@2024-04-22
0.92
A/large-billed crow/Hokkaido/0104B122/2024 H5N1 MP 2.3.4.4b@2024-04-11
A/large-billed crow/Hokkaido/B147/2024 H5N1 MP 2.3.4.4b@2024-04-12
A/goshawk/Gifu/1/2023 H5N1 MP 2.3.4.4b@2023-08-31
A/goshawk/Gifu/1/2023 H5N1 MP 2.3.4.4b@2023-11-23
A/spot-billed duck/Gifu/1118/2023 H5N1 MP 2.3.4.4b@2023-11-18
A/large-billed crow/Hokkaido/0103E088/2023 H5N1 MP 2.3.4.4b@2023-03-30
A/large-billed crow/Hokkaido/0103E089/2023 H5N1 MP 2.3.4.4b@2023-03-30
A/large-billed crow/Hokkaido/4810Z002C/2023 H5N1 MP 2.3.4.4b@2023-10-04
A/Harris’s hawk/Hyogo/HU-FA003/2023 H5N1 MP 2.3.4.4b@2023-12-14
A/large-billed crow/Osaka/HU-FA004/2023 H5N1 MP 2.3.4.4b@2023-12-13
A/red-crowned crane/Hokkaido/20231114001/2023 H5N1 MP 2.3.4.4b@2023-11-14
A/red-crowned crane/Hokkaido/20231115001/2023 H5N1 MP 2.3.4.4b@2023-11-15
A/carrion crow/Hokkaido/B079/2024 H5N1 MP 2.3.4.4b@2024-01-18
A/large-billed crow/Hokkaido/B076/2024 H5N1 MP 2.3.4.4b@2024-01-12
A/large-billed crow/Hokkaido/B078/2024 H5N1 MP 2.3.4.4b@2024-01-17
A/large-billed crow/Hokkaido/B067/2023 H5N1 MP 2.3.4.4b@2023-11-23
A/large-billed crow/Hokkaido/B068/2023 H5N1 MP 2.3.4.4b@2023-11-24
0.82
A/duck/Tottori/NK40/2023 H5N1 MP 2.3.4.4b@2023-11-09
A/large-billed crow/Hokkaido/B093/2024 H5N1 MP 2.3.4.4b@2024-02-07
A/white-tailed eagle/Hokkaido/20240110002/2024 H5N1 MP 2.3.4.4b@2024-01-15
A/large-billed crow/Hokkaido/B114/2024 H5N1 MP 2.3.4.4b@2024-02-26
A/large-billed crow/Hokkaido/B121/2024 H5N1 MP 2.3.4.4b@2024-03-17
A/chicken/Gunma/23A11T/2023 H5N1 MP 2.3.4.4b@2023-12-31
A/chicken/Gunma/23A12T/2023 H5N1 MP 2.3.4.4b@2023-12-31
A/chicken/Yamaguchi/23A1T/2024 H5N1 MP 2.3.4.4b@2024-01-26
A/chicken/Yamaguchi/23A3T/2024 H5N1 MP 2.3.4.4b@2024-01-26
A/goshawk/Gunma/1001A028/2024 H5N1 MP 2.3.4.4b@2024-01-15
0.81
A/chicken/Kagawa/23A1T/2024 H5N1 MP 2.3.4.4b@2024-02-05
A/chicken/Kagawa/23A3T/2024 H5N1 MP 2.3.4.4b@2024-02-05
A/large-billed crow/Osaka/2702A030/2024 H5N1 MP 2.3.4.4b@2024-02-10
A/large-billed crow/Osaka/2702A031/2024 H5N1 MP 2.3.4.4b@2024-02-10
A/large-billed crow/Osaka/2702A032/2024 H5N1 MP 2.3.4.4b@2024-02-10
A/large-billed crow/Osaka/2702A033/2024 H5N1 MP 2.3.4.4b@2024-02-10
A/large-billed crow/Osaka/2702A043/2024 H5N1 MP 2.3.4.4b@2024-02-14
A/large-billed crow/Osaka/2702A044/2024 H5N1 MP 2.3.4.4b@2024-02-15
A/large-billed crow/Osaka/2702A045/2024 H5N1 MP 2.3.4.4b@2024-02-15
A/duck/Korea/D448-N1/2023 H5N1 MP 2.3.4.4b@2023-12-03
A/Northern shoveler/Jeju/D60/2023 H5N1 MP 2.3.4.4b@2023-12-26
0.94
A/chicken/Gifu/23A1T/2024 H5N1 MP 2.3.4.4b@2024-01-05
A/chicken/Gifu/23A7T/2024 H5N1 MP 2.3.4.4b@2024-01-05
A/chicken/Hiroshima/23A2T/2024 H5N1 MP 2.3.4.4b@2024-03-11
A/chicken/Hiroshima/23A6T/2024 H5N1 MP 2.3.4.4b@2024-03-11
A/chicken/Hiroshima/TU10-17/2024 H5N1 MP 2.3.4.4b@2024-03-12
A/chicken/Ibaraki/23A3T/2023 H5N1 MP 2.3.4.4b@2023-11-26
A/chicken/Ibaraki/23A8T/2023 H5N1 MP 2.3.4.4b@2023-11-26
A/chicken/Saitama/23A10C/2023 H5N1 MP 2.3.4.4b@2023-11-29
A/chicken/Saitama/23A6T/2023 H5N1 MP 2.3.4.4b@2023-11-29
A/Eurasian jay/Hokkaido/20240311001/2024 H5N1 MP 2.3.4.4b@2024-03-11
A/Eurasian wigeon/korea/23WS022-22/2023 H5N1 MP 2.3.4.4b@2023-11-27
A/Grus japonensis/Hokkaido/20231106002/2023 H5N1 MP @2023-11-13
A/large-billed crow/Hiroshima/TU10-Ts/ 2024 H5N1 MP 2.3.4.4b@2024-03-12
A/large-billed crow/Hokkaido/0103B117/2024 H5N1 MP 2.3.4.4b@2024-03-22
A/large-billed crow/Hokkaido/0111Q100/2023 H5N1 MP 2.3.4.4b@2023-11-22
A/large-billed crow/Hokkaido/0112Q104/2023 H5N1 MP 2.3.4.4b@2023-12-01
A/large-billed crow/Hokkaido/20231207001/2023 H5N1 MP 2.3.4.4b@2023-12-07
A/large-billed crow/Ishikawa/1702A010/2024 H5N1 MP 2.3.4.4b@2024-02-13
A/large-billed crow/Iwate/0303G012/2024 H5N1 MP 2.3.4.4b@2024-03-13
A/northern pintail/Okayama/331A003/2023 H5N1 MP 2.3.4.4b@2023-11-13
A/peregrine falcon/Hokkaido/4803Z006/2024 H5N1 MP 2.3.4.4b@2024-03-15
A/peregrine falcon/Kumamoto/4301C001/2024 H5N5 MP 2.3.4.4b@2024-01-16
A/whooper swan/Hokkaido/0111E092/2023 H5N1 MP 2.3.4.4b@2023-11-22
A/chicken/Saga/23A1C/2023 H5N1 MP 2.3.4.4b@2023-11-24
A/chicken/Saga/23A2T/2023 H5N1 MP 2.3.4.4b@2023-11-24
0.96
0.74
A/whooper swan/Hokkaido/0112Q105/2023 H5N1 MP 2.3.4.4b@2023-12-04
A/whooper swan/Miyagi/0411B002/2023 H5N1 MP 2.3.4.4b@2023-11-23
0.74
A/large-billed crow/Hokkaido/B155/2024 H5N1 MP 2.3.4.4b@2024-04-26
A/chicken/Chiba/23A10T/2024 H5N1 MP 2.3.4.4b@2024-04-28
A/chicken/Chiba/23A11T/2024 H5N1 MP 2.3.4.4b@2024-04-28
A/chicken/Aomori/22C1C/2023 H5N1 MP 2.3.4.4b@2023-03-24
A/chicken/Aomori/22C2T/2023 H5N1 MP 2.3.4.4b@2023-03-24
A/chicken/Hokkaido/21A3C/2022 H5N1 MP 2.3.4.4b@2022-04-15
A/chicken/Hokkaido/21A4T/2022 H5N1 MP 2.3.4.4b@2022-04-15
A/chicken/Hokkaido/21A5T/2022 H5N1 MP 2.3.4.4b@2022-04-15
1
A/chicken/Hokkaido/21A9T/2022 H5N1 MP 2.3.4.4b@2022-04-15
A/chicken/Hokkaido/I-1/2022 H5N1 MP 2.3.4.4b@2022-04-16
A/chicken/Japan/TU20-2829/2022 H5N1 MP 2.3.4.4b@2022-04-16
1
A/carrion crow/Hokkaido/B081/2024 H5N1 MP 2.3.4.4b@2024-01-26
A/large-billed crow/Hokkaido/B080/2024 H5N1 MP 2.3.4.4b@2024-01-22
0.73
A/Chionis albus/Fildes Peninsula, King George Island/42/2023 H1N1 MP @2023-05-22
0.84
A/goose/Magadan/2272-5/2022 H5N1 MP 2.3.4.4b@2022-10-09
0.93
A/large-billed crow/Fukuoka/4002A007/2023 H5N1 MP 2.3.4.4b@2023-02-17
A/large-billed crow/Hokkaido/B006/2023 H5N1 MP 2.3.4.4b@2023-02-04
A/large-billed crow/Hokkaido/B008/2023 H5N1 MP 2.3.4.4b@2023-03-13
A/large-billed crow/Hokkaido/B049/2023 H5N1 MP 2.3.4.4b@2023-04-11
A/whooper swan/Tochigi/0902C010/2023 H5N1 MP 2.3.4.4b@2023-02-09
A/large-billed crow/Hokkaido/0112P083-2/2022 H5N1 MP 2.3.4.4b@2022-12-05
A/large-billed crow/Hokkaido/B052/2023 H5N1 MP 2.3.4.4b@2023-04-17
A/slaty-backed gull/Hokkaido/0111M114/2022 H5N1 MP 2.3.4.4b@2022-11-16
0.89
A/slaty-backed gull/Hokkaido/0111M111/2022 H5N1 MP 2.3.4.4b@2022-11-06
0.005

## Slide 7
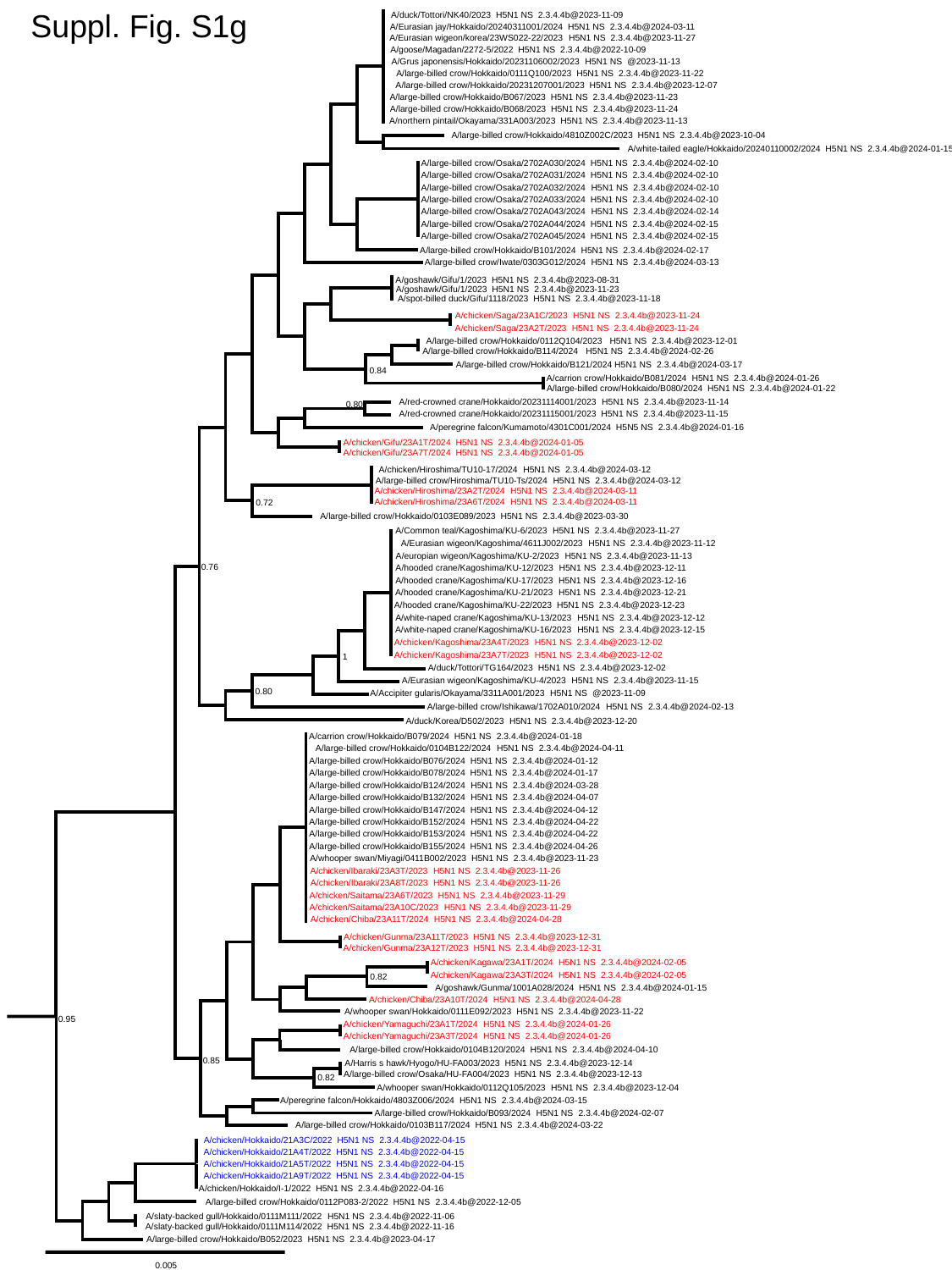

Suppl. Fig. S1g
A/duck/Tottori/NK40/2023 H5N1 NS 2.3.4.4b@2023-11-09
A/Eurasian jay/Hokkaido/20240311001/2024 H5N1 NS 2.3.4.4b@2024-03-11
A/Eurasian wigeon/korea/23WS022-22/2023 H5N1 NS 2.3.4.4b@2023-11-27
A/goose/Magadan/2272-5/2022 H5N1 NS 2.3.4.4b@2022-10-09
A/Grus japonensis/Hokkaido/20231106002/2023 H5N1 NS @2023-11-13
A/large-billed crow/Hokkaido/0111Q100/2023 H5N1 NS 2.3.4.4b@2023-11-22
A/large-billed crow/Hokkaido/20231207001/2023 H5N1 NS 2.3.4.4b@2023-12-07
A/large-billed crow/Hokkaido/B067/2023 H5N1 NS 2.3.4.4b@2023-11-23
A/large-billed crow/Hokkaido/B068/2023 H5N1 NS 2.3.4.4b@2023-11-24
A/northern pintail/Okayama/331A003/2023 H5N1 NS 2.3.4.4b@2023-11-13
A/large-billed crow/Hokkaido/4810Z002C/2023 H5N1 NS 2.3.4.4b@2023-10-04
A/white-tailed eagle/Hokkaido/20240110002/2024 H5N1 NS 2.3.4.4b@2024-01-15
A/large-billed crow/Osaka/2702A030/2024 H5N1 NS 2.3.4.4b@2024-02-10
A/large-billed crow/Osaka/2702A031/2024 H5N1 NS 2.3.4.4b@2024-02-10
A/large-billed crow/Osaka/2702A032/2024 H5N1 NS 2.3.4.4b@2024-02-10
A/large-billed crow/Osaka/2702A033/2024 H5N1 NS 2.3.4.4b@2024-02-10
A/large-billed crow/Osaka/2702A043/2024 H5N1 NS 2.3.4.4b@2024-02-14
A/large-billed crow/Osaka/2702A044/2024 H5N1 NS 2.3.4.4b@2024-02-15
A/large-billed crow/Osaka/2702A045/2024 H5N1 NS 2.3.4.4b@2024-02-15
A/large-billed crow/Hokkaido/B101/2024 H5N1 NS 2.3.4.4b@2024-02-17
A/large-billed crow/Iwate/0303G012/2024 H5N1 NS 2.3.4.4b@2024-03-13
A/goshawk/Gifu/1/2023 H5N1 NS 2.3.4.4b@2023-08-31
A/goshawk/Gifu/1/2023 H5N1 NS 2.3.4.4b@2023-11-23
A/spot-billed duck/Gifu/1118/2023 H5N1 NS 2.3.4.4b@2023-11-18
A/chicken/Saga/23A1C/2023 H5N1 NS 2.3.4.4b@2023-11-24
A/chicken/Saga/23A2T/2023 H5N1 NS 2.3.4.4b@2023-11-24
A/large-billed crow/Hokkaido/0112Q104/2023 H5N1 NS 2.3.4.4b@2023-12-01
A/large-billed crow/Hokkaido/B114/2024 H5N1 NS 2.3.4.4b@2024-02-26
A/large-billed crow/Hokkaido/B121/2024 H5N1 NS 2.3.4.4b@2024-03-17
0.84
A/carrion crow/Hokkaido/B081/2024 H5N1 NS 2.3.4.4b@2024-01-26
A/large-billed crow/Hokkaido/B080/2024 H5N1 NS 2.3.4.4b@2024-01-22
A/red-crowned crane/Hokkaido/20231114001/2023 H5N1 NS 2.3.4.4b@2023-11-14
0.80
A/red-crowned crane/Hokkaido/20231115001/2023 H5N1 NS 2.3.4.4b@2023-11-15
A/peregrine falcon/Kumamoto/4301C001/2024 H5N5 NS 2.3.4.4b@2024-01-16
A/chicken/Gifu/23A1T/2024 H5N1 NS 2.3.4.4b@2024-01-05
A/chicken/Gifu/23A7T/2024 H5N1 NS 2.3.4.4b@2024-01-05
A/chicken/Hiroshima/TU10-17/2024 H5N1 NS 2.3.4.4b@2024-03-12
A/large-billed crow/Hiroshima/TU10-Ts/2024 H5N1 NS 2.3.4.4b@2024-03-12
A/chicken/Hiroshima/23A2T/2024 H5N1 NS 2.3.4.4b@2024-03-11
A/chicken/Hiroshima/23A6T/2024 H5N1 NS 2.3.4.4b@2024-03-11
0.72
A/large-billed crow/Hokkaido/0103E089/2023 H5N1 NS 2.3.4.4b@2023-03-30
A/Common teal/Kagoshima/KU-6/2023 H5N1 NS 2.3.4.4b@2023-11-27
A/Eurasian wigeon/Kagoshima/4611J002/2023 H5N1 NS 2.3.4.4b@2023-11-12
A/europian wigeon/Kagoshima/KU-2/2023 H5N1 NS 2.3.4.4b@2023-11-13
0.76
A/hooded crane/Kagoshima/KU-12/2023 H5N1 NS 2.3.4.4b@2023-12-11
A/hooded crane/Kagoshima/KU-17/2023 H5N1 NS 2.3.4.4b@2023-12-16
A/hooded crane/Kagoshima/KU-21/2023 H5N1 NS 2.3.4.4b@2023-12-21
A/hooded crane/Kagoshima/KU-22/2023 H5N1 NS 2.3.4.4b@2023-12-23
A/white-naped crane/Kagoshima/KU-13/2023 H5N1 NS 2.3.4.4b@2023-12-12
A/white-naped crane/Kagoshima/KU-16/2023 H5N1 NS 2.3.4.4b@2023-12-15
A/chicken/Kagoshima/23A4T/2023 H5N1 NS 2.3.4.4b@2023-12-02
A/chicken/Kagoshima/23A7T/2023 H5N1 NS 2.3.4.4b@2023-12-02
1
A/duck/Tottori/TG164/2023 H5N1 NS 2.3.4.4b@2023-12-02
A/Eurasian wigeon/Kagoshima/KU-4/2023 H5N1 NS 2.3.4.4b@2023-11-15
0.80
A/Accipiter gularis/Okayama/3311A001/2023 H5N1 NS @2023-11-09
A/large-billed crow/Ishikawa/1702A010/2024 H5N1 NS 2.3.4.4b@2024-02-13
A/duck/Korea/D502/2023 H5N1 NS 2.3.4.4b@2023-12-20
A/carrion crow/Hokkaido/B079/2024 H5N1 NS 2.3.4.4b@2024-01-18
A/large-billed crow/Hokkaido/0104B122/2024 H5N1 NS 2.3.4.4b@2024-04-11
A/large-billed crow/Hokkaido/B076/2024 H5N1 NS 2.3.4.4b@2024-01-12
A/large-billed crow/Hokkaido/B078/2024 H5N1 NS 2.3.4.4b@2024-01-17
A/large-billed crow/Hokkaido/B124/2024 H5N1 NS 2.3.4.4b@2024-03-28
A/large-billed crow/Hokkaido/B132/2024 H5N1 NS 2.3.4.4b@2024-04-07
A/large-billed crow/Hokkaido/B147/2024 H5N1 NS 2.3.4.4b@2024-04-12
A/large-billed crow/Hokkaido/B152/2024 H5N1 NS 2.3.4.4b@2024-04-22
A/large-billed crow/Hokkaido/B153/2024 H5N1 NS 2.3.4.4b@2024-04-22
A/large-billed crow/Hokkaido/B155/2024 H5N1 NS 2.3.4.4b@2024-04-26
A/whooper swan/Miyagi/0411B002/2023 H5N1 NS 2.3.4.4b@2023-11-23
A/chicken/Ibaraki/23A3T/2023 H5N1 NS 2.3.4.4b@2023-11-26
A/chicken/Ibaraki/23A8T/2023 H5N1 NS 2.3.4.4b@2023-11-26
A/chicken/Saitama/23A6T/2023 H5N1 NS 2.3.4.4b@2023-11-29
A/chicken/Saitama/23A10C/2023 H5N1 NS 2.3.4.4b@2023-11-29
A/chicken/Chiba/23A11T/2024 H5N1 NS 2.3.4.4b@2024-04-28
A/chicken/Gunma/23A11T/2023 H5N1 NS 2.3.4.4b@2023-12-31
A/chicken/Gunma/23A12T/2023 H5N1 NS 2.3.4.4b@2023-12-31
A/chicken/Kagawa/23A1T/2024 H5N1 NS 2.3.4.4b@2024-02-05
A/chicken/Kagawa/23A3T/2024 H5N1 NS 2.3.4.4b@2024-02-05
0.82
A/goshawk/Gunma/1001A028/2024 H5N1 NS 2.3.4.4b@2024-01-15
A/chicken/Chiba/23A10T/2024 H5N1 NS 2.3.4.4b@2024-04-28
A/whooper swan/Hokkaido/0111E092/2023 H5N1 NS 2.3.4.4b@2023-11-22
0.95
A/chicken/Yamaguchi/23A1T/2024 H5N1 NS 2.3.4.4b@2024-01-26
A/chicken/Yamaguchi/23A3T/2024 H5N1 NS 2.3.4.4b@2024-01-26
A/large-billed crow/Hokkaido/0104B120/2024 H5N1 NS 2.3.4.4b@2024-04-10
0.85
A/Harris s hawk/Hyogo/HU-FA003/2023 H5N1 NS 2.3.4.4b@2023-12-14
A/large-billed crow/Osaka/HU-FA004/2023 H5N1 NS 2.3.4.4b@2023-12-13
0.82
A/whooper swan/Hokkaido/0112Q105/2023 H5N1 NS 2.3.4.4b@2023-12-04
A/peregrine falcon/Hokkaido/4803Z006/2024 H5N1 NS 2.3.4.4b@2024-03-15
A/large-billed crow/Hokkaido/B093/2024 H5N1 NS 2.3.4.4b@2024-02-07
A/large-billed crow/Hokkaido/0103B117/2024 H5N1 NS 2.3.4.4b@2024-03-22
A/chicken/Hokkaido/21A3C/2022 H5N1 NS 2.3.4.4b@2022-04-15
A/chicken/Hokkaido/21A4T/2022 H5N1 NS 2.3.4.4b@2022-04-15
A/chicken/Hokkaido/21A5T/2022 H5N1 NS 2.3.4.4b@2022-04-15
A/chicken/Hokkaido/21A9T/2022 H5N1 NS 2.3.4.4b@2022-04-15
A/chicken/Hokkaido/I-1/2022 H5N1 NS 2.3.4.4b@2022-04-16
A/large-billed crow/Hokkaido/0112P083-2/2022 H5N1 NS 2.3.4.4b@2022-12-05
A/slaty-backed gull/Hokkaido/0111M111/2022 H5N1 NS 2.3.4.4b@2022-11-06
A/slaty-backed gull/Hokkaido/0111M114/2022 H5N1 NS 2.3.4.4b@2022-11-16
A/large-billed crow/Hokkaido/B052/2023 H5N1 NS 2.3.4.4b@2023-04-17
0.005
