# Supplementary material for: Phylogenetic and Pathogenic Analysis of H5N1 and H5N6 High Pathogenicity Avian Influenza Virus Isolated from Poultry Farms (Layer and Broiler Chickens) in Japan in the 2023/2024 Season
Source: Viruses. 2024 Dec 20;16(12):1956. doi: 10.3390/v16121956 (PMC11680161; doi:10.3390/v16121956)
Supplement: Supplementary file 1 [file viruses-16-01956-s001.zip › Suppl. Figure S2.pptx]

## Slide 1
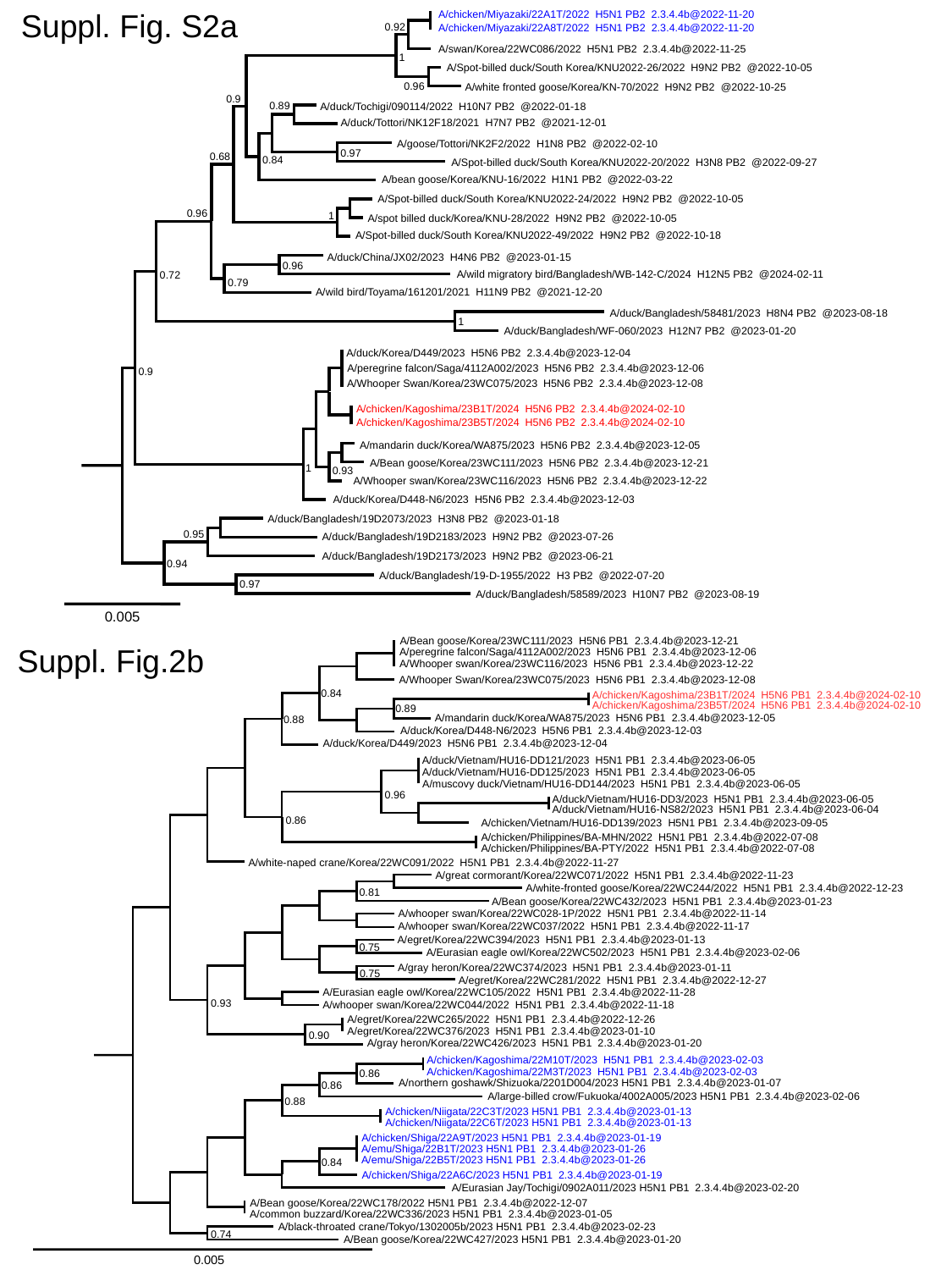

Suppl. Fig. S2a
A/chicken/Miyazaki/22A1T/2022 H5N1 PB2 2.3.4.4b@2022-11-20
0.92
A/chicken/Miyazaki/22A8T/2022 H5N1 PB2 2.3.4.4b@2022-11-20
A/swan/Korea/22WC086/2022 H5N1 PB2 2.3.4.4b@2022-11-25
1
A/Spot-billed duck/South Korea/KNU2022-26/2022 H9N2 PB2 @2022-10-05
0.96
A/white fronted goose/Korea/KN-70/2022 H9N2 PB2 @2022-10-25
0.9
0.89
A/duck/Tochigi/090114/2022 H10N7 PB2 @2022-01-18
A/duck/Tottori/NK12F18/2021 H7N7 PB2 @2021-12-01
A/goose/Tottori/NK2F2/2022 H1N8 PB2 @2022-02-10
0.97
0.68
0.84
A/Spot-billed duck/South Korea/KNU2022-20/2022 H3N8 PB2 @2022-09-27
A/bean goose/Korea/KNU-16/2022 H1N1 PB2 @2022-03-22
A/Spot-billed duck/South Korea/KNU2022-24/2022 H9N2 PB2 @2022-10-05
0.96
1
A/spot billed duck/Korea/KNU-28/2022 H9N2 PB2 @2022-10-05
A/Spot-billed duck/South Korea/KNU2022-49/2022 H9N2 PB2 @2022-10-18
A/duck/China/JX02/2023 H4N6 PB2 @2023-01-15
0.96
A/wild migratory bird/Bangladesh/WB-142-C/2024 H12N5 PB2 @2024-02-11
0.72
0.79
A/wild bird/Toyama/161201/2021 H11N9 PB2 @2021-12-20
A/duck/Bangladesh/58481/2023 H8N4 PB2 @2023-08-18
1
A/duck/Bangladesh/WF-060/2023 H12N7 PB2 @2023-01-20
A/duck/Korea/D449/2023 H5N6 PB2 2.3.4.4b@2023-12-04
A/peregrine falcon/Saga/4112A002/2023 H5N6 PB2 2.3.4.4b@2023-12-06
0.9
A/Whooper Swan/Korea/23WC075/2023 H5N6 PB2 2.3.4.4b@2023-12-08
A/chicken/Kagoshima/23B1T/2024 H5N6 PB2 2.3.4.4b@2024-02-10
A/chicken/Kagoshima/23B5T/2024 H5N6 PB2 2.3.4.4b@2024-02-10
A/mandarin duck/Korea/WA875/2023 H5N6 PB2 2.3.4.4b@2023-12-05
A/Bean goose/Korea/23WC111/2023 H5N6 PB2 2.3.4.4b@2023-12-21
1
0.93
A/Whooper swan/Korea/23WC116/2023 H5N6 PB2 2.3.4.4b@2023-12-22
A/duck/Korea/D448-N6/2023 H5N6 PB2 2.3.4.4b@2023-12-03
A/duck/Bangladesh/19D2073/2023 H3N8 PB2 @2023-01-18
0.95
A/duck/Bangladesh/19D2183/2023 H9N2 PB2 @2023-07-26
A/duck/Bangladesh/19D2173/2023 H9N2 PB2 @2023-06-21
0.94
A/duck/Bangladesh/19-D-1955/2022 H3 PB2 @2022-07-20
0.97
A/duck/Bangladesh/58589/2023 H10N7 PB2 @2023-08-19
0.005
A/Bean goose/Korea/23WC111/2023 H5N6 PB1 2.3.4.4b@2023-12-21
Suppl. Fig.2b
A/peregrine falcon/Saga/4112A002/2023 H5N6 PB1 2.3.4.4b@2023-12-06
A/Whooper swan/Korea/23WC116/2023 H5N6 PB1 2.3.4.4b@2023-12-22
A/Whooper Swan/Korea/23WC075/2023 H5N6 PB1 2.3.4.4b@2023-12-08
0.84
A/chicken/Kagoshima/23B1T/2024 H5N6 PB1 2.3.4.4b@2024-02-10
A/chicken/Kagoshima/23B5T/2024 H5N6 PB1 2.3.4.4b@2024-02-10
0.89
A/mandarin duck/Korea/WA875/2023 H5N6 PB1 2.3.4.4b@2023-12-05
0.88
A/duck/Korea/D448-N6/2023 H5N6 PB1 2.3.4.4b@2023-12-03
A/duck/Korea/D449/2023 H5N6 PB1 2.3.4.4b@2023-12-04
A/duck/Vietnam/HU16-DD121/2023 H5N1 PB1 2.3.4.4b@2023-06-05
A/duck/Vietnam/HU16-DD125/2023 H5N1 PB1 2.3.4.4b@2023-06-05
A/muscovy duck/Vietnam/HU16-DD144/2023 H5N1 PB1 2.3.4.4b@2023-06-05
0.96
A/duck/Vietnam/HU16-DD3/2023 H5N1 PB1 2.3.4.4b@2023-06-05
A/duck/Vietnam/HU16-NS82/2023 H5N1 PB1 2.3.4.4b@2023-06-04
0.86
A/chicken/Vietnam/HU16-DD139/2023 H5N1 PB1 2.3.4.4b@2023-09-05
A/chicken/Philippines/BA-MHN/2022 H5N1 PB1 2.3.4.4b@2022-07-08
A/chicken/Philippines/BA-PTY/2022 H5N1 PB1 2.3.4.4b@2022-07-08
A/white-naped crane/Korea/22WC091/2022 H5N1 PB1 2.3.4.4b@2022-11-27
A/great cormorant/Korea/22WC071/2022 H5N1 PB1 2.3.4.4b@2022-11-23
A/white-fronted goose/Korea/22WC244/2022 H5N1 PB1 2.3.4.4b@2022-12-23
0.81
A/Bean goose/Korea/22WC432/2023 H5N1 PB1 2.3.4.4b@2023-01-23
A/whooper swan/Korea/22WC028-1P/2022 H5N1 PB1 2.3.4.4b@2022-11-14
A/whooper swan/Korea/22WC037/2022 H5N1 PB1 2.3.4.4b@2022-11-17
A/egret/Korea/22WC394/2023 H5N1 PB1 2.3.4.4b@2023-01-13
0.75
A/Eurasian eagle owl/Korea/22WC502/2023 H5N1 PB1 2.3.4.4b@2023-02-06
A/gray heron/Korea/22WC374/2023 H5N1 PB1 2.3.4.4b@2023-01-11
0.75
A/egret/Korea/22WC281/2022 H5N1 PB1 2.3.4.4b@2022-12-27
A/Eurasian eagle owl/Korea/22WC105/2022 H5N1 PB1 2.3.4.4b@2022-11-28
0.93
A/whooper swan/Korea/22WC044/2022 H5N1 PB1 2.3.4.4b@2022-11-18
A/egret/Korea/22WC265/2022 H5N1 PB1 2.3.4.4b@2022-12-26
A/egret/Korea/22WC376/2023 H5N1 PB1 2.3.4.4b@2023-01-10
0.90
A/gray heron/Korea/22WC426/2023 H5N1 PB1 2.3.4.4b@2023-01-20
A/chicken/Kagoshima/22M10T/2023 H5N1 PB1 2.3.4.4b@2023-02-03
A/chicken/Kagoshima/22M3T/2023 H5N1 PB1 2.3.4.4b@2023-02-03
0.86
A/northern goshawk/Shizuoka/2201D004/2023 H5N1 PB1 2.3.4.4b@2023-01-07
0.86
A/large-billed crow/Fukuoka/4002A005/2023 H5N1 PB1 2.3.4.4b@2023-02-06
0.88
A/chicken/Niigata/22C3T/2023 H5N1 PB1 2.3.4.4b@2023-01-13
A/chicken/Niigata/22C6T/2023 H5N1 PB1 2.3.4.4b@2023-01-13
A/chicken/Shiga/22A9T/2023 H5N1 PB1 2.3.4.4b@2023-01-19
A/emu/Shiga/22B1T/2023 H5N1 PB1 2.3.4.4b@2023-01-26
A/emu/Shiga/22B5T/2023 H5N1 PB1 2.3.4.4b@2023-01-26
0.84
A/chicken/Shiga/22A6C/2023 H5N1 PB1 2.3.4.4b@2023-01-19
A/Eurasian Jay/Tochigi/0902A011/2023 H5N1 PB1 2.3.4.4b@2023-02-20
A/Bean goose/Korea/22WC178/2022 H5N1 PB1 2.3.4.4b@2022-12-07
A/common buzzard/Korea/22WC336/2023 H5N1 PB1 2.3.4.4b@2023-01-05
A/black-throated crane/Tokyo/1302005b/2023 H5N1 PB1 2.3.4.4b@2023-02-23
0.74
A/Bean goose/Korea/22WC427/2023 H5N1 PB1 2.3.4.4b@2023-01-20
0.005

## Slide 2
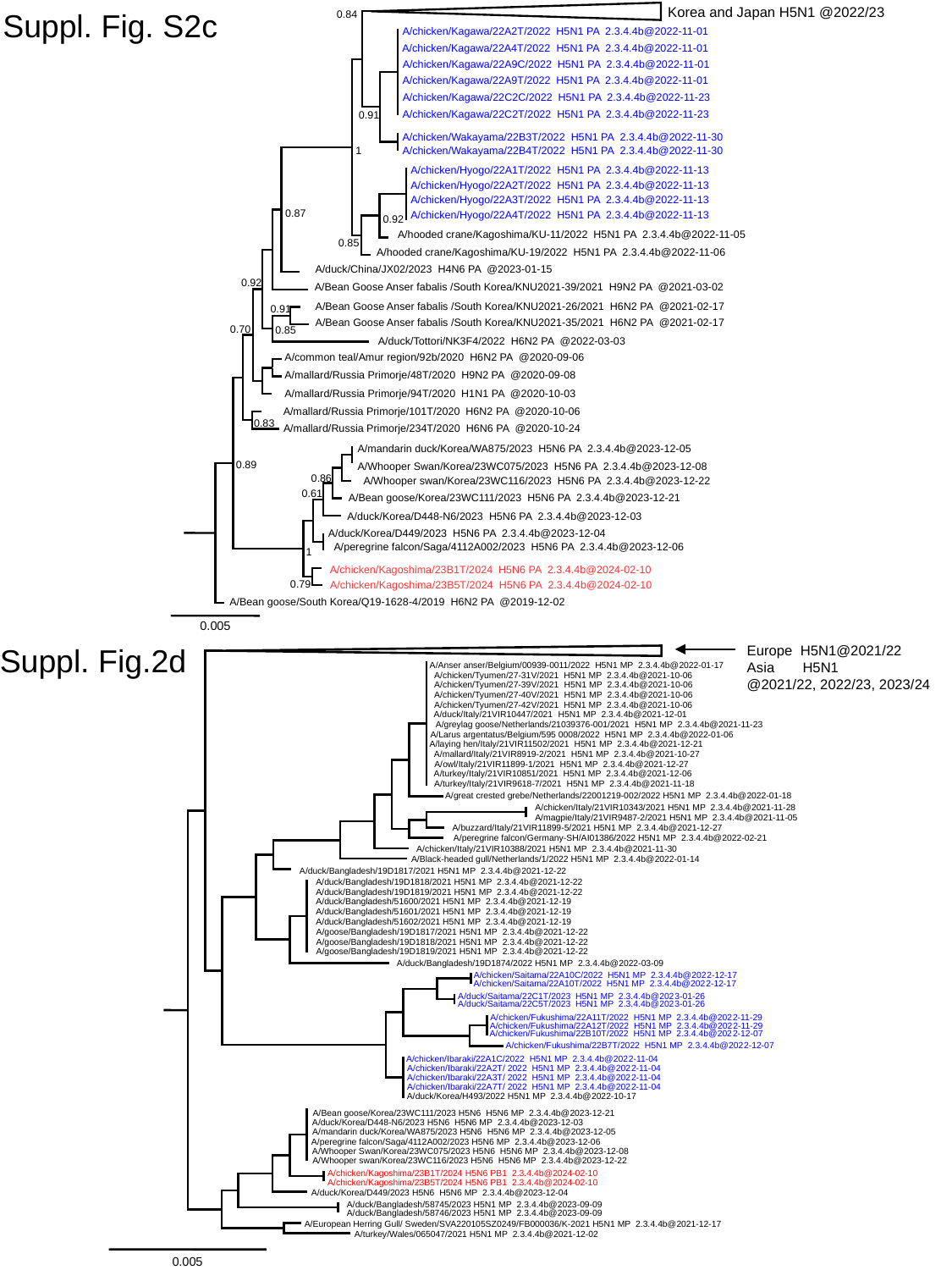

Suppl. Fig. S2c
Korea and Japan H5N1 @2022/23
0.84
A/chicken/Kagawa/22A2T/2022 H5N1 PA 2.3.4.4b@2022-11-01
A/chicken/Kagawa/22A4T/2022 H5N1 PA 2.3.4.4b@2022-11-01
A/chicken/Kagawa/22A9C/2022 H5N1 PA 2.3.4.4b@2022-11-01
A/chicken/Kagawa/22A9T/2022 H5N1 PA 2.3.4.4b@2022-11-01
A/chicken/Kagawa/22C2C/2022 H5N1 PA 2.3.4.4b@2022-11-23
A/chicken/Kagawa/22C2T/2022 H5N1 PA 2.3.4.4b@2022-11-23
0.91
A/chicken/Wakayama/22B3T/2022 H5N1 PA 2.3.4.4b@2022-11-30
A/chicken/Wakayama/22B4T/2022 H5N1 PA 2.3.4.4b@2022-11-30
1
A/chicken/Hyogo/22A1T/2022 H5N1 PA 2.3.4.4b@2022-11-13
A/chicken/Hyogo/22A2T/2022 H5N1 PA 2.3.4.4b@2022-11-13
A/chicken/Hyogo/22A3T/2022 H5N1 PA 2.3.4.4b@2022-11-13
0.87
A/chicken/Hyogo/22A4T/2022 H5N1 PA 2.3.4.4b@2022-11-13
0.92
A/hooded crane/Kagoshima/KU-11/2022 H5N1 PA 2.3.4.4b@2022-11-05
0.85
A/hooded crane/Kagoshima/KU-19/2022 H5N1 PA 2.3.4.4b@2022-11-06
A/duck/China/JX02/2023 H4N6 PA @2023-01-15
0.92
A/Bean Goose Anser fabalis /South Korea/KNU2021-39/2021 H9N2 PA @2021-03-02
A/Bean Goose Anser fabalis /South Korea/KNU2021-26/2021 H6N2 PA @2021-02-17
0.91
A/Bean Goose Anser fabalis /South Korea/KNU2021-35/2021 H6N2 PA @2021-02-17
0.70
0.85
A/duck/Tottori/NK3F4/2022 H6N2 PA @2022-03-03
A/common teal/Amur region/92b/2020 H6N2 PA @2020-09-06
A/mallard/Russia Primorje/48T/2020 H9N2 PA @2020-09-08
A/mallard/Russia Primorje/94T/2020 H1N1 PA @2020-10-03
A/mallard/Russia Primorje/101T/2020 H6N2 PA @2020-10-06
0.83
A/mallard/Russia Primorje/234T/2020 H6N6 PA @2020-10-24
A/mandarin duck/Korea/WA875/2023 H5N6 PA 2.3.4.4b@2023-12-05
0.89
A/Whooper Swan/Korea/23WC075/2023 H5N6 PA 2.3.4.4b@2023-12-08
0.86
A/Whooper swan/Korea/23WC116/2023 H5N6 PA 2.3.4.4b@2023-12-22
0.61
A/Bean goose/Korea/23WC111/2023 H5N6 PA 2.3.4.4b@2023-12-21
A/duck/Korea/D448-N6/2023 H5N6 PA 2.3.4.4b@2023-12-03
A/duck/Korea/D449/2023 H5N6 PA 2.3.4.4b@2023-12-04
A/peregrine falcon/Saga/4112A002/2023 H5N6 PA 2.3.4.4b@2023-12-06
1
A/chicken/Kagoshima/23B1T/2024 H5N6 PA 2.3.4.4b@2024-02-10
0.79
A/chicken/Kagoshima/23B5T/2024 H5N6 PA 2.3.4.4b@2024-02-10
A/Bean goose/South Korea/Q19-1628-4/2019 H6N2 PA @2019-12-02
0.005
Suppl. Fig.2d
Europe H5N1@2021/22
Asia H5N1
@2021/22, 2022/23, 2023/24
A/Anser anser/Belgium/00939-0011/2022 H5N1 MP 2.3.4.4b@2022-01-17
A/chicken/Tyumen/27-31V/2021 H5N1 MP 2.3.4.4b@2021-10-06
A/chicken/Tyumen/27-39V/2021 H5N1 MP 2.3.4.4b@2021-10-06
A/chicken/Tyumen/27-40V/2021 H5N1 MP 2.3.4.4b@2021-10-06
A/chicken/Tyumen/27-42V/2021 H5N1 MP 2.3.4.4b@2021-10-06
A/duck/Italy/21VIR10447/2021 H5N1 MP 2.3.4.4b@2021-12-01
A/greylag goose/Netherlands/21039376-001/2021 H5N1 MP 2.3.4.4b@2021-11-23
A/Larus argentatus/Belgium/595 0008/2022 H5N1 MP 2.3.4.4b@2022-01-06
A/laying hen/Italy/21VIR11502/2021 H5N1 MP 2.3.4.4b@2021-12-21
A/mallard/Italy/21VIR8919-2/2021 H5N1 MP 2.3.4.4b@2021-10-27
A/owl/Italy/21VIR11899-1/2021 H5N1 MP 2.3.4.4b@2021-12-27
A/turkey/Italy/21VIR10851/2021 H5N1 MP 2.3.4.4b@2021-12-06
A/turkey/Italy/21VIR9618-7/2021 H5N1 MP 2.3.4.4b@2021-11-18
A/great crested grebe/Netherlands/22001219-002/2022 H5N1 MP 2.3.4.4b@2022-01-18
A/chicken/Italy/21VIR10343/2021 H5N1 MP 2.3.4.4b@2021-11-28
A/magpie/Italy/21VIR9487-2/2021 H5N1 MP 2.3.4.4b@2021-11-05
A/buzzard/Italy/21VIR11899-5/2021 H5N1 MP 2.3.4.4b@2021-12-27
A/peregrine falcon/Germany-SH/AI01386/2022 H5N1 MP 2.3.4.4b@2022-02-21
A/chicken/Italy/21VIR10388/2021 H5N1 MP 2.3.4.4b@2021-11-30
A/Black-headed gull/Netherlands/1/2022 H5N1 MP 2.3.4.4b@2022-01-14
A/duck/Bangladesh/19D1817/2021 H5N1 MP 2.3.4.4b@2021-12-22
A/duck/Bangladesh/19D1818/2021 H5N1 MP 2.3.4.4b@2021-12-22
A/duck/Bangladesh/19D1819/2021 H5N1 MP 2.3.4.4b@2021-12-22
A/duck/Bangladesh/51600/2021 H5N1 MP 2.3.4.4b@2021-12-19
A/duck/Bangladesh/51601/2021 H5N1 MP 2.3.4.4b@2021-12-19
A/duck/Bangladesh/51602/2021 H5N1 MP 2.3.4.4b@2021-12-19
A/goose/Bangladesh/19D1817/2021 H5N1 MP 2.3.4.4b@2021-12-22
A/goose/Bangladesh/19D1818/2021 H5N1 MP 2.3.4.4b@2021-12-22
A/goose/Bangladesh/19D1819/2021 H5N1 MP 2.3.4.4b@2021-12-22
A/duck/Bangladesh/19D1874/2022 H5N1 MP 2.3.4.4b@2022-03-09
A/chicken/Saitama/22A10C/2022 H5N1 MP 2.3.4.4b@2022-12-17
A/chicken/Saitama/22A10T/2022 H5N1 MP 2.3.4.4b@2022-12-17
A/duck/Saitama/22C1T/2023 H5N1 MP 2.3.4.4b@2023-01-26
A/duck/Saitama/22C5T/2023 H5N1 MP 2.3.4.4b@2023-01-26
A/chicken/Fukushima/22A11T/2022 H5N1 MP 2.3.4.4b@2022-11-29
A/chicken/Fukushima/22A12T/2022 H5N1 MP 2.3.4.4b@2022-11-29
A/chicken/Fukushima/22B10T/2022 H5N1 MP 2.3.4.4b@2022-12-07
A/chicken/Fukushima/22B7T/2022 H5N1 MP 2.3.4.4b@2022-12-07
A/chicken/Ibaraki/22A1C/2022 H5N1 MP 2.3.4.4b@2022-11-04
A/chicken/Ibaraki/22A2T/ 2022 H5N1 MP 2.3.4.4b@2022-11-04
A/chicken/Ibaraki/22A3T/ 2022 H5N1 MP 2.3.4.4b@2022-11-04
A/chicken/Ibaraki/22A7T/ 2022 H5N1 MP 2.3.4.4b@2022-11-04
A/duck/Korea/H493/2022 H5N1 MP 2.3.4.4b@2022-10-17
A/Bean goose/Korea/23WC111/2023 H5N6 H5N6 MP 2.3.4.4b@2023-12-21
A/duck/Korea/D448-N6/2023 H5N6 H5N6 MP 2.3.4.4b@2023-12-03
A/mandarin duck/Korea/WA875/2023 H5N6 H5N6 MP 2.3.4.4b@2023-12-05
A/peregrine falcon/Saga/4112A002/2023 H5N6 MP 2.3.4.4b@2023-12-06
A/Whooper Swan/Korea/23WC075/2023 H5N6 H5N6 MP 2.3.4.4b@2023-12-08
A/Whooper swan/Korea/23WC116/2023 H5N6 H5N6 MP 2.3.4.4b@2023-12-22
A/chicken/Kagoshima/23B1T/2024 H5N6 PB1 2.3.4.4b@2024-02-10
A/chicken/Kagoshima/23B5T/2024 H5N6 PB1 2.3.4.4b@2024-02-10
A/duck/Korea/D449/2023 H5N6 H5N6 MP 2.3.4.4b@2023-12-04
A/duck/Bangladesh/58745/2023 H5N1 MP 2.3.4.4b@2023-09-09
A/duck/Bangladesh/58746/2023 H5N1 MP 2.3.4.4b@2023-09-09
A/European Herring Gull/ Sweden/SVA220105SZ0249/FB000036/K-2021 H5N1 MP 2.3.4.4b@2021-12-17
A/turkey/Wales/065047/2021 H5N1 MP 2.3.4.4b@2021-12-02
0.005
