# Supplementary material for: Phylogenetic and Pathogenic Analysis of H5N1 and H5N6 High Pathogenicity Avian Influenza Virus Isolated from Poultry Farms (Layer and Broiler Chickens) in Japan in the 2023/2024 Season
Source: Viruses. 2024 Dec 20;16(12):1956. doi: 10.3390/v16121956 (PMC11680161; doi:10.3390/v16121956)
Supplement: Supplementary file 1 [file viruses-16-01956-s001.zip › Suppl. Figure S3.pptx]

## Slide 1
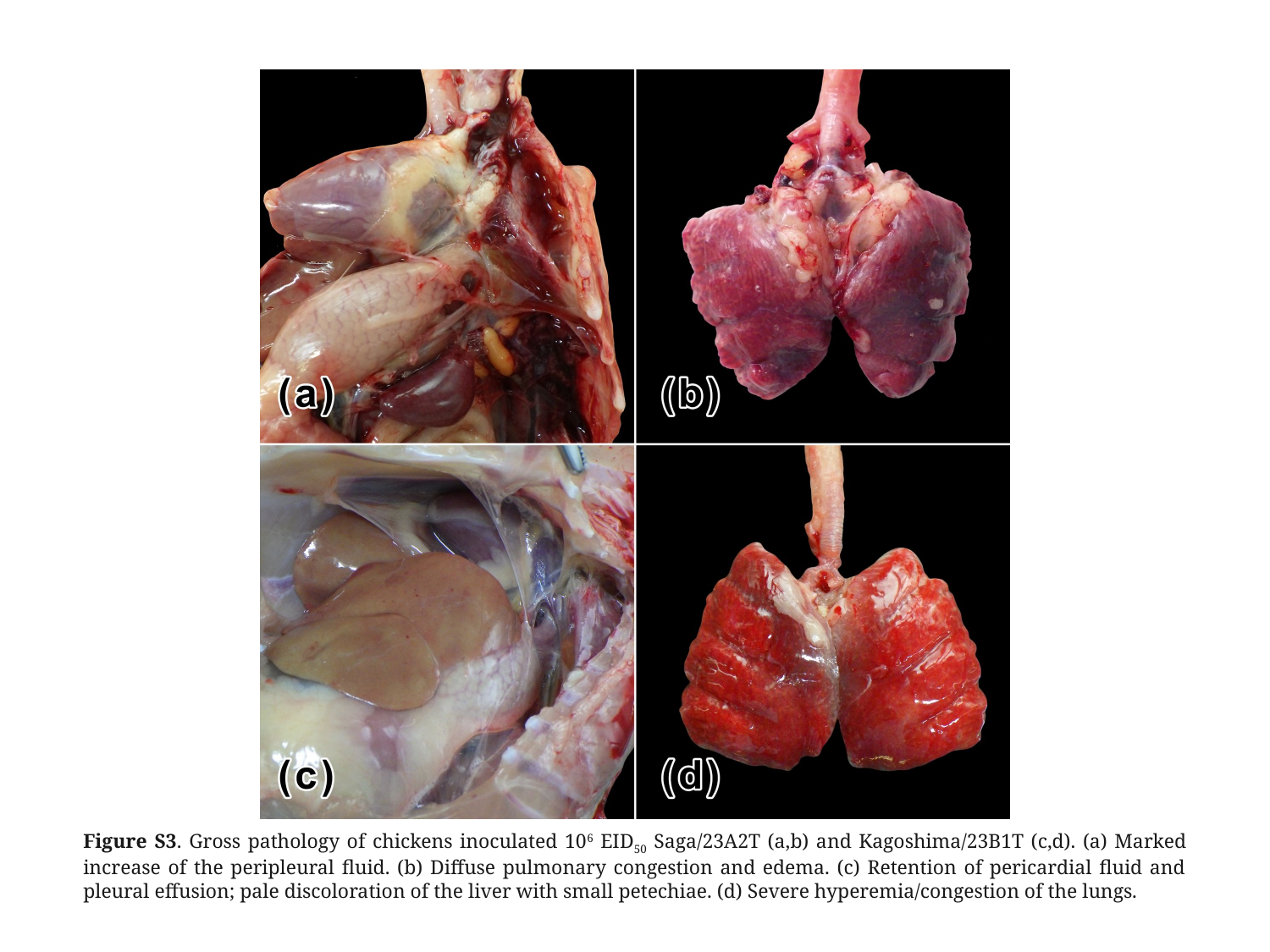

Figure S3. Gross pathology of chickens inoculated 106 EID50 Saga/23A2T (a,b) and Kagoshima/23B1T (c,d). (a) Marked increase of the peripleural fluid. (b) Diffuse pulmonary congestion and edema. (c) Retention of pericardial fluid and pleural effusion; pale discoloration of the liver with small petechiae. (d) Severe hyperemia/congestion of the lungs.
