# Supplementary material for: Phylogenetic and Pathogenic Analysis of H5N1 and H5N6 High Pathogenicity Avian Influenza Virus Isolated from Poultry Farms (Layer and Broiler Chickens) in Japan in the 2023/2024 Season
Source: Viruses. 2024 Dec 20;16(12):1956. doi: 10.3390/v16121956 (PMC11680161; doi:10.3390/v16121956)
Supplement: Supplementary file 1 [file viruses-16-01956-s001.zip › Suppl. Figure S4.pptx]

## Slide 1
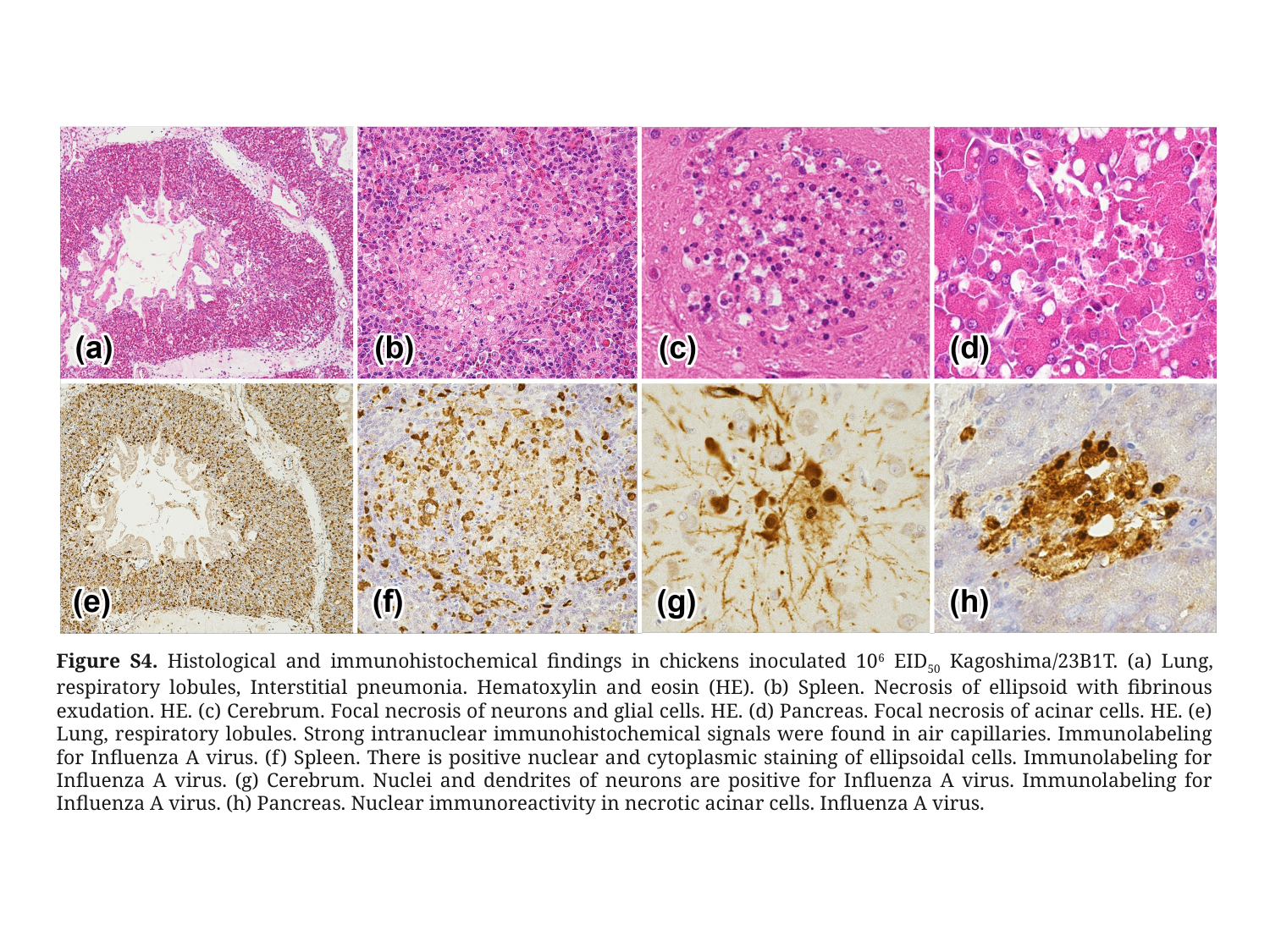

Figure S4. Histological and immunohistochemical findings in chickens inoculated 106 EID50 Kagoshima/23B1T. (a) Lung, respiratory lobules, Interstitial pneumonia. Hematoxylin and eosin (HE). (b) Spleen. Necrosis of ellipsoid with fibrinous exudation. HE. (c) Cerebrum. Focal necrosis of neurons and glial cells. HE. (d) Pancreas. Focal necrosis of acinar cells. HE. (e) Lung, respiratory lobules. Strong intranuclear immunohistochemical signals were found in air capillaries. Immunolabeling for Influenza A virus. (f) Spleen. There is positive nuclear and cytoplasmic staining of ellipsoidal cells. Immunolabeling for Influenza A virus. (g) Cerebrum. Nuclei and dendrites of neurons are positive for Influenza A virus. Immunolabeling for Influenza A virus. (h) Pancreas. Nuclear immunoreactivity in necrotic acinar cells. Influenza A virus.
